# The TBC1D15 Oncoprotein Controls Stem Cell Self-Renewal through Destabilization of the Numb-p53 Complex

**DOI:** 10.1371/journal.pone.0057312

**Published:** 2013-02-27

**Authors:** Douglas E. Feldman, Chialin Chen, Vasu Punj, Keigo Machida

**Affiliations:** 1 Department of Molecular Microbiology and Immunology, University of Southern California, Keck School of Medicine, Los Angeles, California, United States of America; 2 Bioinformatics Core, Norris Comprehensive Cancer Center at University of Southern California and Division of Hematology, University of Southern California, Keck School of Medicine, Los Angeles, California, United States of America; 3 Southern California Research Center for ALPD and Cirrhosis, University of Southern California, Keck School of Medicine, Los Angeles, California, United States of America; Inserm, U1052, UMR 5286, France

## Abstract

Stem cell populations are maintained through self-renewing divisions in which one daughter cell commits to a specific fate while the other retains the multipotent characteristics of its parent. The p53 tumor suppressor, in conjunction with its interacting partner protein Numb, preserves this asymmetry and functions as a vital barrier against the unchecked expansion of tumor stem cell pools; however, little is known about the biological control of the Numb-p53 interaction. We show here that Numb and p53 are the constituents of a high molecular mass complex, which is disintegrated upon activation of aPKCζ, a Numb kinase. Using large-scale affinity purification and tandem mass spectrometry, we identify TBC1D15 as a Numb-associated protein and demonstrate that its amino-terminal domain disengages p53 from Numb, triggering p53 proteolysis and promoting self-renewal and pluripotency. Cellular levels of TBC1D15 are diminished upon acute nutrient deprivation through autophagy-mediated degradation, indicating that TBC1D15 serves as a conduit through which cellular metabolic status is linked to self-renewal. The profound deregulation of TBC1D15 expression exhibited in a diverse array of patient tumors underscores its proposed function as an oncoprotein.

## Introduction

Highly malignant tumor-initiating stem-like cells (TISCs) have been identified as rare subpopulations within a broad array of solid tumors and hematological malignancies arising from diverse tissue types [1]–[4]. The potent capacity of TISCs to seed and regenerate tumors following serial engraftment and reisolation, as well as a resistance to radiation and conventional chemotherapy, stand as defining features of these cells, however we do not yet have a coherent understanding of TISC origins or of the deficiencies in control which account for their unchecked proliferation and clinical intractability [5], [6].

TISCs share key features with embryonic stem cells (ESCs) present within preimplantation blastocyst stage embryos, including the expression of a core pluripotency-associated transcription factor (TF) network [7]–[11], but in contrast to ESCs, TISCs fail to properly control the self-renewing mode of cell division that is a fundamental property of stem cells. In untransformed stem cells, self-renewal typically occurs through asymmetric cell division, in which one daughter cell retains the multipotent progenitor status of its parent while the other cell commits to a specialized cell fate. TISCs exhibit a striking loss of this intrinsic asymmetry, leading to the implementation of stem cell-associated gene expression programs in both progeny and subsequently to unchecked expansion of the progenitor cell pool [12]–[14].

Misregulation of stem cell division can occur as the result of genetic lesions targeting diverse aspects of cellular homeostasis, from chromatin structure [15] to signal transduction cascades activated by the extracellular ligands epidermal growth factor (EGF) [12] and Hedgehog [16]. Notwithstanding this apparent complexity, many of these pathways converge functionally to inactivate the p53 tumor suppressor [17], which serves as a critical barrier to cellular reprogramming to the pluripotent state and stem cell proliferation [18]. This aspect of p53 function may be carried out in part through direct genetic repression of pluripotency-associated TF network components [19].

Inactivation of p53 in tumor stem cells also leads to a loss of cell polarity and to aberrant execution of self-renewing divisions [12]. Cells deficient in p53 fail to correctly localize Numb, a cell fate determinant that has itself been identified independently as a tumor suppressor [20], [21]. In polarized epithelial progenitor cells and in mitotic stem cells, Numb is distributed asymmetrically and segregates into the daughter cell that undergoes differentiation. Intriguingly, Numb also interacts directly with p53, protecting it from ubiquitin-mediated proteolysis triggered by the MDM2 E3 ubiquitin ligase [22]. As a dual regulator of cell polarity and p53 stability, Numb is exceptionally well positioned to control stem cell self-renewal. However, little is known about the composition, regulation or functional significance of the Numb-p53 complex. Prompted by these questions, we conducted a biochemical analysis of this complex in murine hepatocellular carcinoma-derived TISCs and identified an interacting protein, TBC1D15, which destabilizes the Numb-p53 complex and is itself subject to autophagy-mediated degradation upon nutrient depletion. These findings highlight a potential role for TBC1D15 in connecting cellular energy status and stem cell self-renewal.

## Results

**Figure 1:**
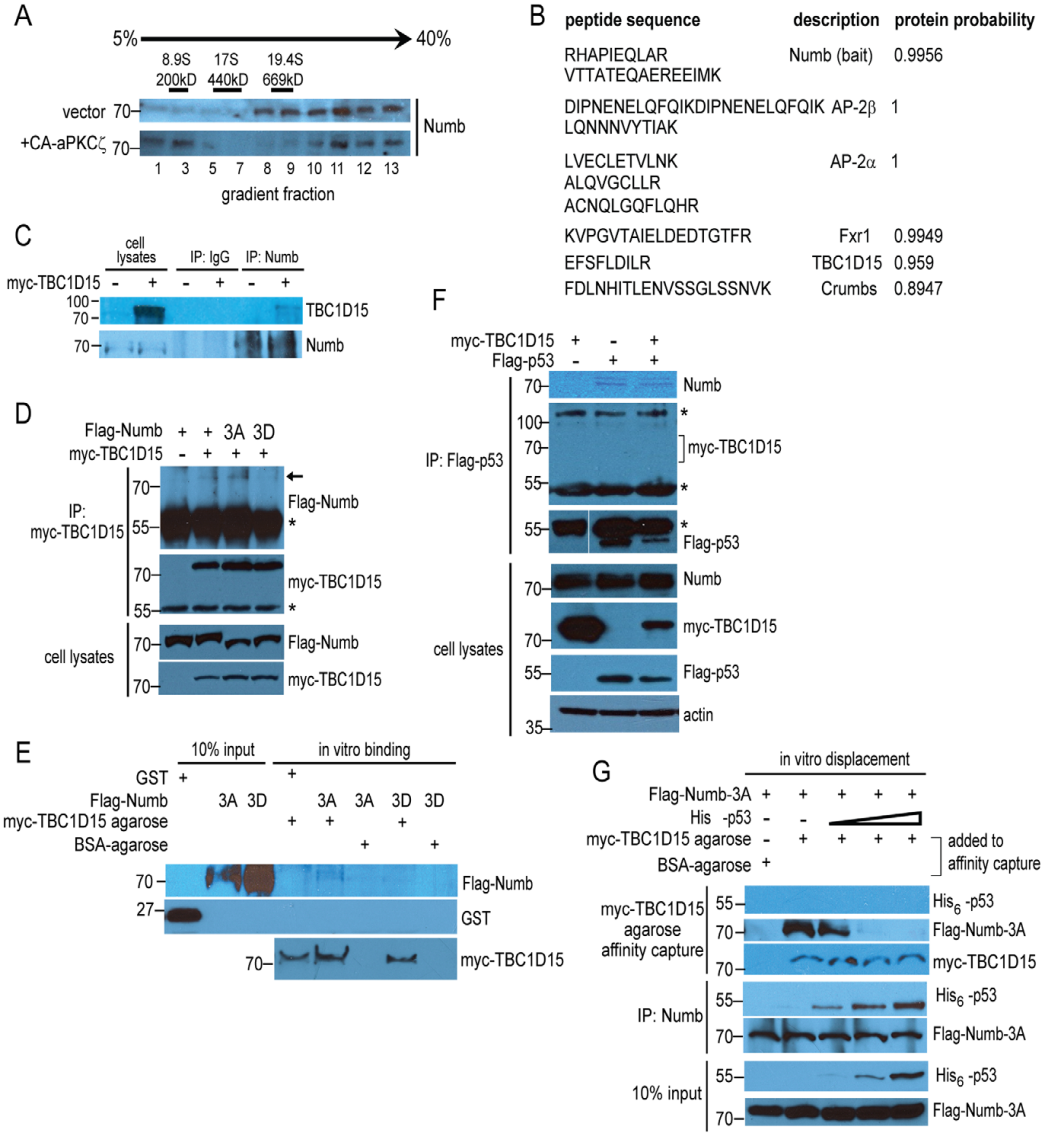
Identification of a high molecular mass Numb complex containing TBC1D15. (A) Cytoplasmic lysates prepared from CD133+/CD49f+ mouse liver TISCs stably transduced with empty vector or a vector encoding constitutively active aPKCζ (CA-aPKCζ) were loaded separately onto 5–40% continuous sucrose density gradients and resolved by centrifugation, followed by SDS-PAGE and immunoblotting with anti-Numb antisera. The sedimentation positions of molecular mass standards analyzed in parallel on an identical gradient are indicated above the immunoblots. (B) Identification of candidate Numb-interacting proteins. High molecular mass Numb complexes were immunoaffinity purified from pooled sucrose gradient fractions 8–13, followed by polypeptide identification using LC-MS/MS. High-confidence interacting proteins identified from this purification strategy are shown. (C) Lysates prepared from TISCs transfected with either empty vector or with vector encoding myc-TBC1D15 were subjected to immunoprecipitation using anti-Numb agarose resin. Immunoprecipitates and cytoplasmic lysates corresponding to 15% of the input to the immunoprecipitation were analyzed by immunoblotting. (D) TISCs were transfected with the indicated variants of Flag-Numb alone or together with myc-TBC1D15, then lysed and immunoprecipitated with anti-myc antibody, followed by immunoblotting. Asterisks indicate cross-reacting immunoglobulin signals. Lysates represent 10% of the input volume to the immunoprecipitations. (E) Interaction of purified myc-TBC1D15 and Numb in vitro. GST control protein or recombinant, purified Flag-Numb-3A or Flag-Numb-3D were mixed with BSA-coated control resin or with myc-TBC1D15 agarose, as indicated. Agarose resin was recovered by centrifugation after extensive washing and bound proteins were resolved by SDS-PAGE followed by immunoblotting. (F) HEK-293A cells were transfected with the indicated combinations of Flag-p53 and myc-TBC1D15, followed by lysis and immunoprecipitation using anti-Flag antibody. Input lysates and immunoprecipitated proteins were analyzed by immunoblotting using the indicated antibodies. Asterisks indicate cross-reacting immunoglobulin signals. (G) In vitro binding displacement assay. Purified Flag-Numb-3A was incubated in buffer alone or with increasing amounts of purified His_6_-p53. A portion of the binding reaction was subjected to immunoprecipitation using anti-Numb agarose, while the remaining volume was mixed with either BSA-agarose or myc-TBC1D15 agarose, followed by extensive washing. Protein inputs and agarose resin-bound proteins were resolved by SDS-PAGE and analyzed by immunoblotting.

We set out to examine biochemically the composition of the Numb-p53 complex and to determine whether misregulation of this complex contributes to the aberrant execution of self-renewal in tumor stem cells. Analysis by continuous sucrose density gradient centrifugation of cytoplasmic lysates prepared from CD133+/CD49f+ murine liver TISCs [23] revealed that endogenous Numb is a constituent of high molecular mass (>700 kDa) complexes (Figure 1A). Numb is phosphorylated in vivo by atypical protein kinase C (aPKCζ) [24], [25]. We found that the expression of a constitutively active form of aPKCζ (CA- aPKCζ) disengaged a portion of Numb from its association with a high molecular mass complex, resulting in the appearance of a lower molecular mass species which migrated near the top of the gradient (Figure 1A).

To gain additional insight into the composition and regulation of Numb complexes in TISCs, we conducted a large-scale immunoaffinity purification of endogenous Numb from pooled sucrose gradient fractions (fractions 8–13), followed by liquid chromatography-tandem mass spectrometry (LC-MS/MS) to identify associated polypeptides. By employing stringent scoring metrics [26], we identified several high-confidence interacting proteins (Figure 1B), including AP-2α and AP-2β, components of the clathrin-associated endocytic adaptor complex shown previously to associate with Numb [27], [28]. We additionally identified in association with Numb a single peptide from TBC1D15, which has been implicated in vesicle trafficking to the lysosome and in the regulation of autophagy [29], [30]. To validate this interaction, we first expressed recombinant, myc-tagged TBC1D15 (myc-TBC1D15) in TISCs, followed by lysis and immunoprecipitation of endogenous Numb. As shown in Figure 1C, myc-TBC1D15 associated specifically with Numb following stringent washes. A reciprocal immunoprecipitation of myc-TBC1D15, co-expressed in TISCs with Flag-Numb, substantiated the interaction (Figure 1D). Using an antibody raised against TBC1D15, we carried out reciprocal immunoprecipitations of the endogenous, untagged proteins and confirmed the interaction of Numb and TBC1D15 in two additional cell lines, PIL-4 hepatoblasts [31] and HeLa cervical carcinoma cells (Figure S1). The Flag-Numb-3A mutant, which carries alanine substitutions in the three major aPKCζ serine phosphorylation sites [25], associated stably with myc-TBC1D15, while a mutant form of Numb harboring the phosphomimetic residue aspartic acid in each of these positions (Flag-Numb-3D) did not detectably interact with myc-TBC1D15 (Figure 1D). These findings suggest that aPKCζ -directed phosphorylation of Numb regulates its affinity for TBC1D15, as it does for the Numb-interacting protein integrin [25].

To examine whether Numb can interact directly with TBC1D15, we incubated highly purified, monomeric Flag-Numb-3A or -3D variants in the presence of agarose beads coated with either bovine serum albumin (BSA) as a control or myc-TBC1D15 immunopurified from mammalian cell lysates (Figure S2). Following stringent washes, Flag-Numb-3A was recovered specifically in association with myc-TBC1D15, while an interaction with the Flag-Numb-3D mutant was once again not detected (Figure 1E), supporting the proposal that TBC1D15 interacts directly with a form of Numb that has not been phosphorylated by aPKCζ at critical serine residues.

As Numb can also interact directly with p53 and shields it from proteolysis by MDM2 [22], we asked whether p53, TBC1D15 and Numb assemble together into a single macromolecular complex. We evaluated the interaction of endogenous Numb with Flag-p53 and myc-TBC1D15 in HEK-293A cells, a cell line widely utilized for robust expression of recombinant proteins and the study of protein-protein interactions. While endogenous Numb was recovered following immunoprecipitation of Flag-p53, recombinant myc-TBC1D15 was not detected in the same immunoprecipitates (Figure 1F). To examine more closely the relationship between TBC1D15, p53 and Numb, we developed an in vitro binding competition assay in which a fixed concentration of Flag-Numb-3A was incubated alone or in the presence of increasing concentrations of purified, hexahistidine-tagged p53 (His_6_-p53). This initial incubation was then mixed with BSA- or myc-TBC1D15-coated agarose resin or used separately as the input to a Numb immunoprecipitation. In the absence of His_6_-p53, Flag-Numb-3A was recovered in myc-TBC1D15 agarose precipitates (Figure 1G). However, the addition of increasing amounts of His_6_-p53 to the initial incubation decreased the yield of Flag-Numb-3A recovered with myc-TBC1D15 (Figure 1G, upper panels) while producing a corresponding increase in His_6_-p53 associated with Numb (Figure 1G, middle panels). No His_6_-p53 could be detected in association with myc-TBC1D15, supporting the proposal that TBC1D15 and p53 form distinct complexes with Numb.

**Figure 2:**
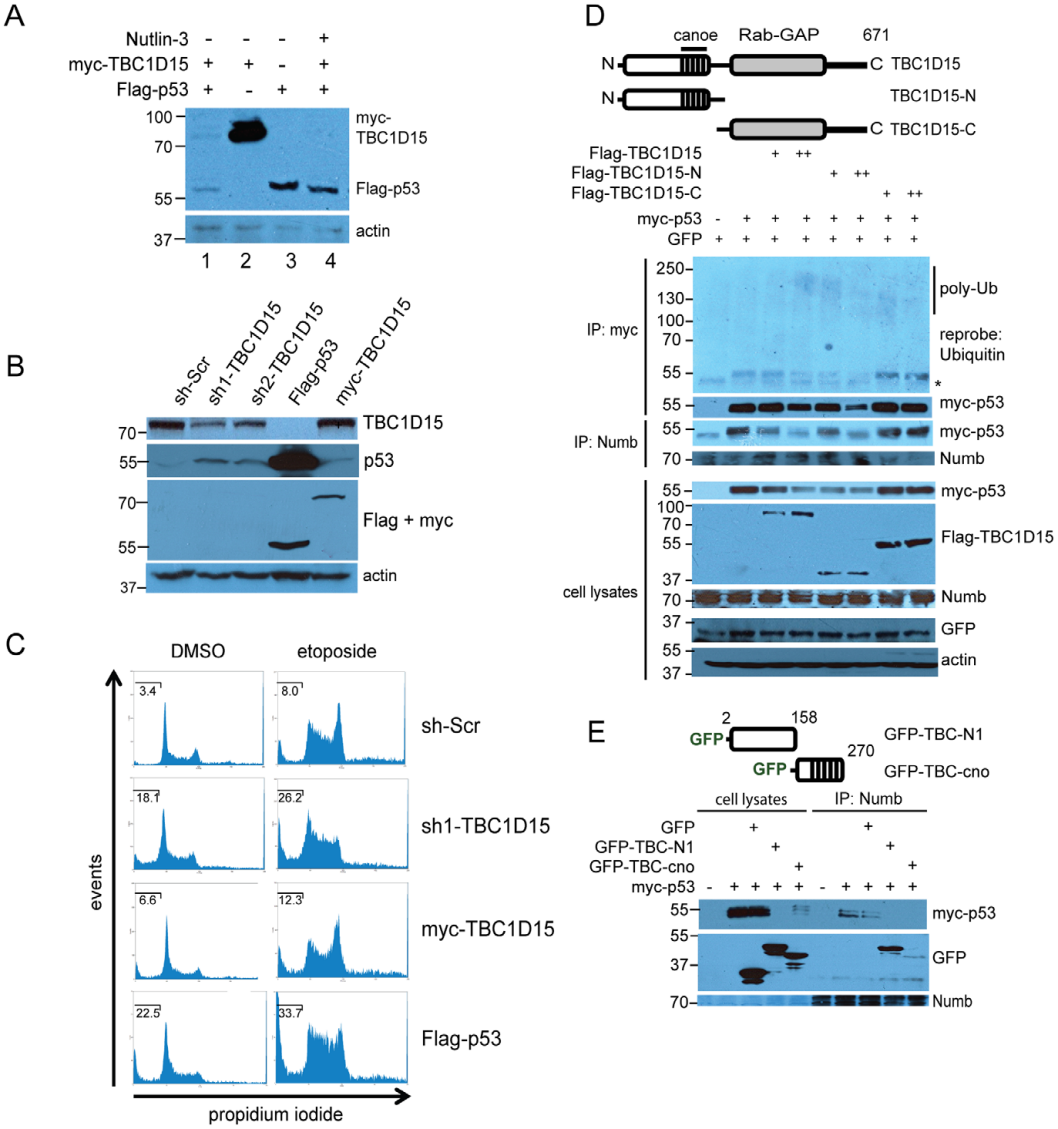
The Numb-binding domain of TBC1D15 targets p53 for proteolysis. (A) Following transfection with the indicated expression vectors, HEK-293A cells were exposed to vehicle or Nutlin-3 (10 μM) for 16 hr as indicated, then lysed and analyzed by immunoblotting. (B) Depletion of endogenous *TBC1D15* increases steady-state levels of p53. HEK-293A cells were stably transduced with lentivirus vectors encoding a non-targeting, scrambled shRNA control (sh-*Scr*) or two different shRNAs targeting *TBC1D15*, or transfected with Flag-p53 or myc-TBC1D15, followed by preparation of lysates, SDS-PAGE and immunoblotting using the indicated antibodies. (C) Depletion of TBC1D15 sensitizes cells to apoptosis. HEK-293A cells harboring a non-targeting shRNA or sh1-*TBC1D15,* or expressing either myc-TBC1D15 or Flag-p53, were treated with DMSO vehicle or with etoposide (34 μM, 16 hr) followed by propidium iodide staining and FACS analysis. The percentage of sub-G1 DNA content is indicated in each histogram. (D) The amino-terminal domain of TBC1D15 targets p53 for proteolysis. Myc-p53 was expressed in HEK-293A cells with empty vector or with the indicated Flag-TBC1D15 constructs. Lysates were immunoprecipitated using either anti-Numb or anti-myc agarose. GFP was co-transfected as an internal control for transfection efficiency. (E) Functional dissection of the TBC1D15 amino-terminal domain in p53 destabilization. Myc-p53 was transfected with GFP or with the indicated GFP-TBC1D15 fusions, followed by lysis, Numb immunoprecipitation and immunoblotting. Lysates represent 10% of the input volume used in the immunoprecipitation.

In the above experiments, steady-state levels of Flag-p53 were decreased in the presence of co-transfected myc-TBC1D15 (Figure 1F, bottom panels). Indeed, expression of myc-TBC1D15 diminished levels of co-expressed Flag-p53 (Figure 2A, lanes 1 and 3). Exposure of cells to Nutlin-3 blocked this effect, suggesting that myc-TBC1D15 stimulates the MDM2-mediated proteolysis of Flag-p53 (Figure 2A, lanes 1 and 4). A striking reciprocal inhibition of myc-TBC1D15 by Flag-p53 was also readily observed in these experiments. Stable expression of two distinct short hairpin RNAs (shRNAs) to deplete *TBC1D15* increased levels of endogenous p53 in a manner that corresponded closely with the degree of *TBC1D15* depletion (Figure 2B). Cells depleted for *TBC1D15* were accordingly sensitized to apoptosis induced by the type-II topoisomerase inhibitor etoposide, as determined by increased sub-G1 DNA content (sh-*Scr*: 8.0+/−0.34%; sh-*TBC1D15*∶26.2+/−1.76%, *P*<0.05) (Figure 2C).

TBC1D15 is comprised of two distinct structural domains: a carboxyl-terminal GTPase-activating protein (GAP) that has been shown to act on the Rab7 GTPase [29] and a functionally uncharacterized amino-terminal domain. We expressed Flag-tagged variants of each domain individually with myc-p53, and found that the TBC1CD15 amino-terminal domain (Flag-TBC1D15-N) recapitulated inhibition of myc-p53 (Figure 2D). In these experiments, destabilization and polyubiquitination of myc-p53 corresponded closely with the extent of its displacement from Numb (Figure 2D).

Sequence analysis of the TBC1D15 amino-terminal domain revealed a 50 amino acid region containing significant homology to the Drosophila protein Canoe (Figure S3), which regulates the localization of cell-fate determinants during asymmetric neuroblast division [32], [33]. Coexpression of myc-p53 with GFP fusion proteins containing either the Canoe homology region (TBC-cno, amino acids 159–270) or the TBC1D15 amino-terminal peptide region (TBC-N1, amino acids 2–158) revealed that both fusions diminished steady-state levels of myc-p53, while GFP alone had no effect on the stability of myc-p53 or on its association with Numb (Figure 2E). In these experiments, a greater proportion of the GFP-TBC-N1 fusion associated stably with Numb (Figure 2E), suggesting that sequence or higher order structural motifs within this region of TBC1D15 direct binding to Numb.

**Figure 3:**
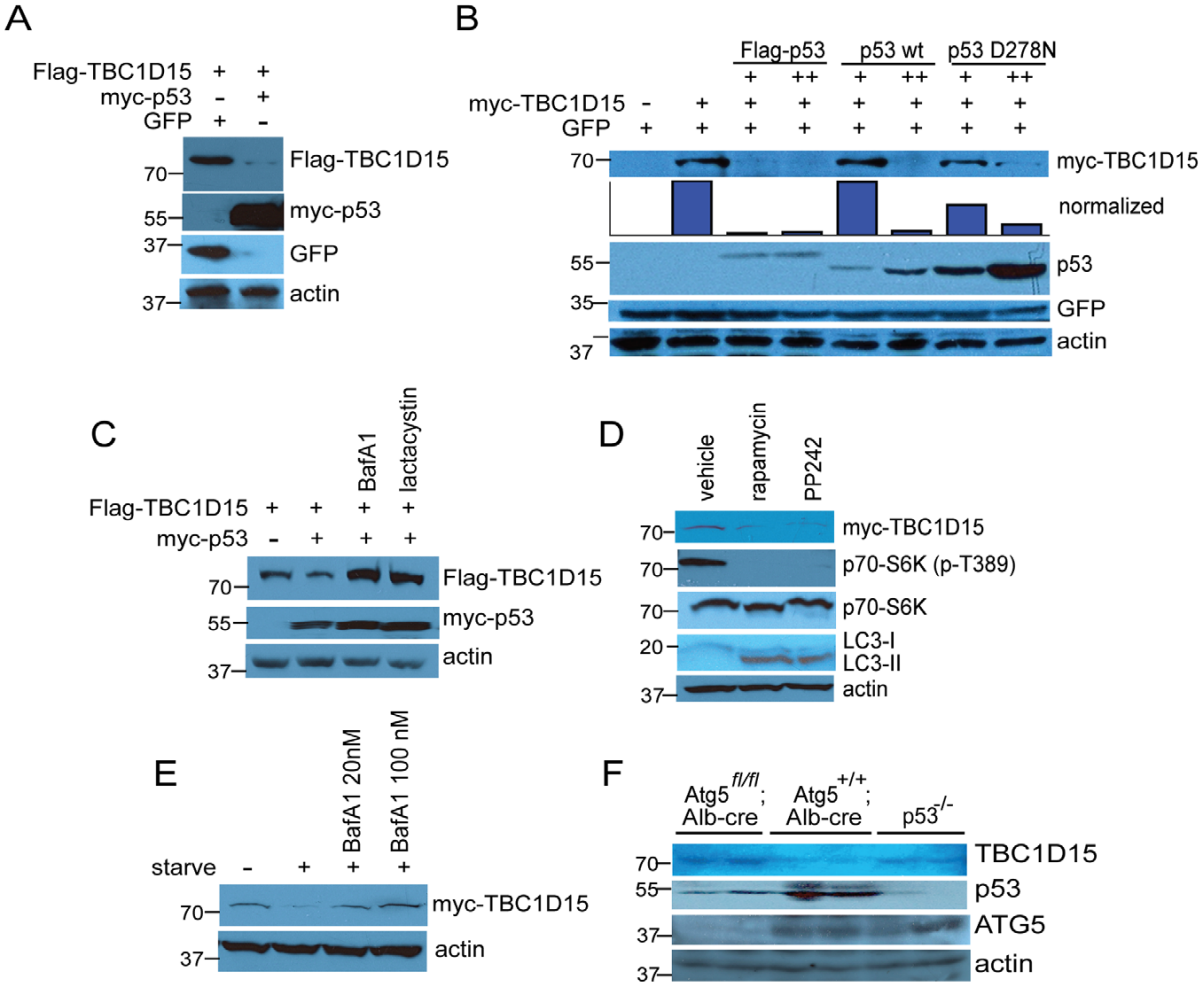
Starvation- and p53-induced degradation of TBC1D15. (A) Flag- TBC1D15 was expressed alone or with myc-p53 as indicated, followed by cell lysis and analysis by immunoblotting. (B) p53 transactivation function mediates destabilization of myc-TBC1D15. HEK-293A cells were transfected with myc-TBC1D15 and either empty vector or Flag-p53 (human), wild-type murine p53 or the transactivation-deficient point mutant p53D278N, followed by lysis and immunoblotting. (C) Cells were transfected with Flag-TBC1D15 and either empty vector or myc-p53, followed by exposure for 6 hr to autophagy inhibitor Bafilomycin A1 (100 nM) or proteasome inhibitor lactacystin (5 μM). (D) HEK-293A cells expressing myc-TBC1D15 were exposed for 6 hr to DMSO vehicle or to the mTOR inhibitors rapamycin (100 nM) or PP242 (1 μM), then lysed and analyzed by immunoblotting. (E) Cells were transfected with myc-TBC1D15 and cultured in complete media or shifted to glucose- and amino acid-free media for 6 hr in the absence or presence of Bafilomycin A1, as indicated. (F) Elevated levels of TBC1D15 in *Atg5*- and *p53*-deficient livers. Livers were surgically resected from p53-deficient mice or from mice with hepatocyte-specific deletion of *Atg5* (*Atg5^fl^* ^/fl^; *Alb-cre*) or littermate controls. Liver lysates were resolved by SDS-PAGE and analyzed by immunoblotting.

Evidence from the TBC1D15 and p53 co-expression experiments (Figures 1F and 2A) suggested that the antagonistic relationship between these proteins is bidirectional. Indeed, expression of myc-p53 diminished the levels of co-transfected Flag-TBC1D15 (Figure 3A), and a transactivation-impaired point mutant of murine p53 (D278N) [34] was less efficient than wild-type p53 in destabilizing myc-TBC1D15 (Figure 3B), suggesting that transcriptional induction of downstream effectors by p53 is required to inhibit TBC1D15. We note, however, that this mutation may also produce a conformational change in p53 that destabilizes its interaction with other protein partners and hence could impair extra-transcriptional functions of p53.

Proteomic approaches have identified TBC1D15 as a substrate for polyubiquitination [35] and as a component of an autophagy-related protein network through its interaction with members of the ATG8/MAP-LC3 protein family [30]. We therefore asked whether p53-mediated destabilization of TBC1D15 proceeds through either of these degradative pathways. Exposure of HEK-293A cells to the proteasome inhibitor lactacystin or to Bafilomycin A1, an inhibitor of autophagic flux to the lysosome, stabilized Flag-TBC1D15 in the presence of myc-p53 (Figure 3C). Conversely, induction of autophagy by two distinct small molecule inhibitors of the mTOR kinase, rapamycin and PP242, diminished phosphorylation at Thr-389 of the mTOR substrate p70 S6K and decreased steady-state levels of myc-TBC1D15 (Figure 3D). Acute nutrient deprivation likewise diminished myc-TBC1D15, which was rescued by treatment with Bafilomycin A1 in a dosage-sensitive manner (Figure 3E). In line with these observations, livers isolated from *Atg5 ^fl/fl^; Alb-cre* mice, in which autophagy is blocked in hepatocytes as a result of the biallelic deletion of *Atg5* coding sequences, exhibited a significant accumulation of TBC1D15, an effect also observed in livers obtained from *p53^−/−^* mice (Figure 3F).

While human TBC1D15 is polyubiquitinated on two acceptor lysine residues (K90 and K103) [35], substitution of both corresponding sites to arginine, which cannot accept ubiquitin, in murine TBC1D15 failed to stabilize Flag-TBC1D15 or Flag-TBC1D15-N in the presence of myc-p53 (Figure S4), underscoring a central role for autophagic degradation in the control of TBC1D15 stability and suggesting that the stabilization of Flag-TBC1D15 by lactacystin is an indirect effect.

Interestingly, shRNA-mediated depletion of *TBC1D15* (Figure S5) resulted in increased steady-state levels of autophagosomes, as determined by immunoblotting for the cleaved, lipidated form of LC3 (LC3-II)(Figure S6A), while enforced expression of Flag-TBC1D15 or Flag-TBC-N decreased levels of LC3-II, consistent with a previous report demonstrating that TBC1D15 opposes autophagic activity. Depletion of *TBC1D15* likewise resulted in the induction of DRAM, a p53 target gene encoding a lysosomal protein that induces autophagy, and stimulated expression of the catabolic genes and the p53 effectors SESN2, SCO2 and TIGAR (Figure S6B). In accordance with these findings, metabolic flux analysis of cells depleted for *TBC1D15* revealed enhanced respiratory capacity and increased oxygen consumption rate, while enforced expression of TBC1D15 decreased these parameters and stimulated basal glycolytic flux, as determined by measurement of the extracellular acidification rate (Figures S6C and S6D).

**Figure 4:**
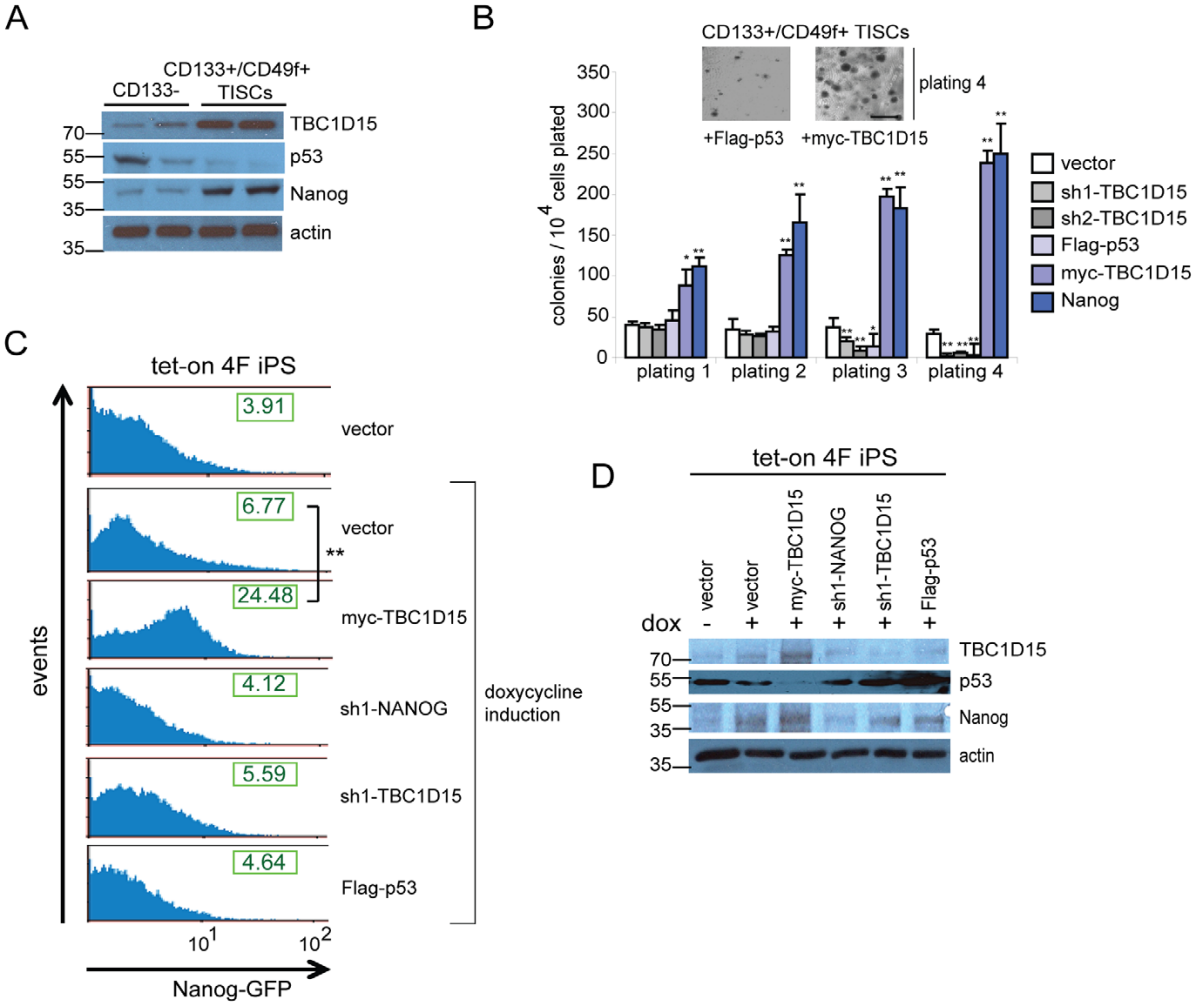
TBC1D15 promotes self-renewal and reprogramming to pluripotency. (A) The expression of TBC1D15, p53 and Nanog was evaluated by immunblotting of cell lysates prepared from CD133- hepatocytes or CD133+/CD49f+ TISCs. (B) Methylcellulose colony formation assay. Murine TISCs stably expressing the indicated transgenes or lentivirus-encoded shRNAs were plated in triplicate in methylcellulose media and cultured for one week prior to colony scoring. Cells were then resuspended, counted and replated for a total of four platings (7,14, 21 and 28 days). Inset, images of representative colonies after four platings are shown for the indicated cell lines. Scale bar, 500 μm. (C) TBC1D15 stimulates reprogramming efficiency. NGFP2 doxycycline-inducible pluripotent stem cells (iPSCs) stably expressing the indicated transgenes or transduced with the indicated shRNAs were either untreated or exposed to doxycycline (2 μg/mL) for 21 days to induce reprogramming. Cells were subsequently harvested and analyzed by FACS. The calculated mean fluorescence intensity of the Nanog-GFP reporter is shown for each plot. (D) Immunoblot analysis of NGFP2 clones. Lysates were prepared from NGFP2 cells stably expressing the indicated vectors following 21 days exposure to vehicle (lane 1) or doxycycline, then resolved by SDS-PAGE and analyzed by immunoblotting using the indicated antibodies.

Given the above indications that TBC1D15 antagonizes p53, we sought to examine the function of TBC1D15 in self-renewal and pluripotency. A comparison of TBC1D15 levels in two independent isolates of murine CD133- hepatocytes and CD133+/CD49f+ liver TISCs revealed increased expression of TBC1D15 in TISCs and a correspondence with the self-renewal factor Nanog (Figure 4A). Colony formation in vitro by stem cells cultured in low-adhesion methylcellulose media serves as a robust functional assay to monitor self-renewal capacity [36], [37]. Enforced expression of myc-TBC1D15 in murine TISCs stimulated colony-formation to a similar extent as expression of the pluripotency factor Nanog, while Flag-p53 or shRNA-mediated depletion of *TBC1D15* () suppressed colony formation following serial replatings (Figure 4B), supporting a role for TBC1D15 in self-renewal. Prompted by these findings, we carried out reprogramming assays using NGFP2 iPS mouse embryonic fibroblasts (MEFs), which harbor stably integrated, doxycycline-inducible transgenes encoding the reprogramming factors Oct4, Sox2, Klf4 and c-Myc [38] together with a Nanog-GFP reporter to enable the quantitative assessment of reprogramming efficiency [39]. Following a 21-day exposure of NGFP2 MEFs to doxycycline, NGFP2 vector control cells exhibited increased mean Nanog-GFP fluorescence intensity relative to uninduced controls (6.77+−/1.7% vs. 3.91+/−0.3%, *P*<0.05) (Figure 4C). Strikingly, stable expression of myc-TBC1D15 during the reprogramming process increased the mean Nanog-GFP fluorescence intensity (24.48+/−3.6%, *P*<0.01 relative to doxycycline-treated controls), while Flag-p53 or shRNAs targeting either *TBC1D15* or *NANOG* modestly decreased Nanog-GFP induction (Figure 4C). Mirroring these findings, immunoblot analysis of lysates prepared from NGFP2 MEFs following a 21-day exposure to doxycycline confirmed that stable expression of TBC1D15 induced Nanog expression with a corresponding decrease in p53 levels (Figure 4D). These results are in line with a previous report that can p53 directly suppress the expression of Nanog [40].

**Figure 5:**
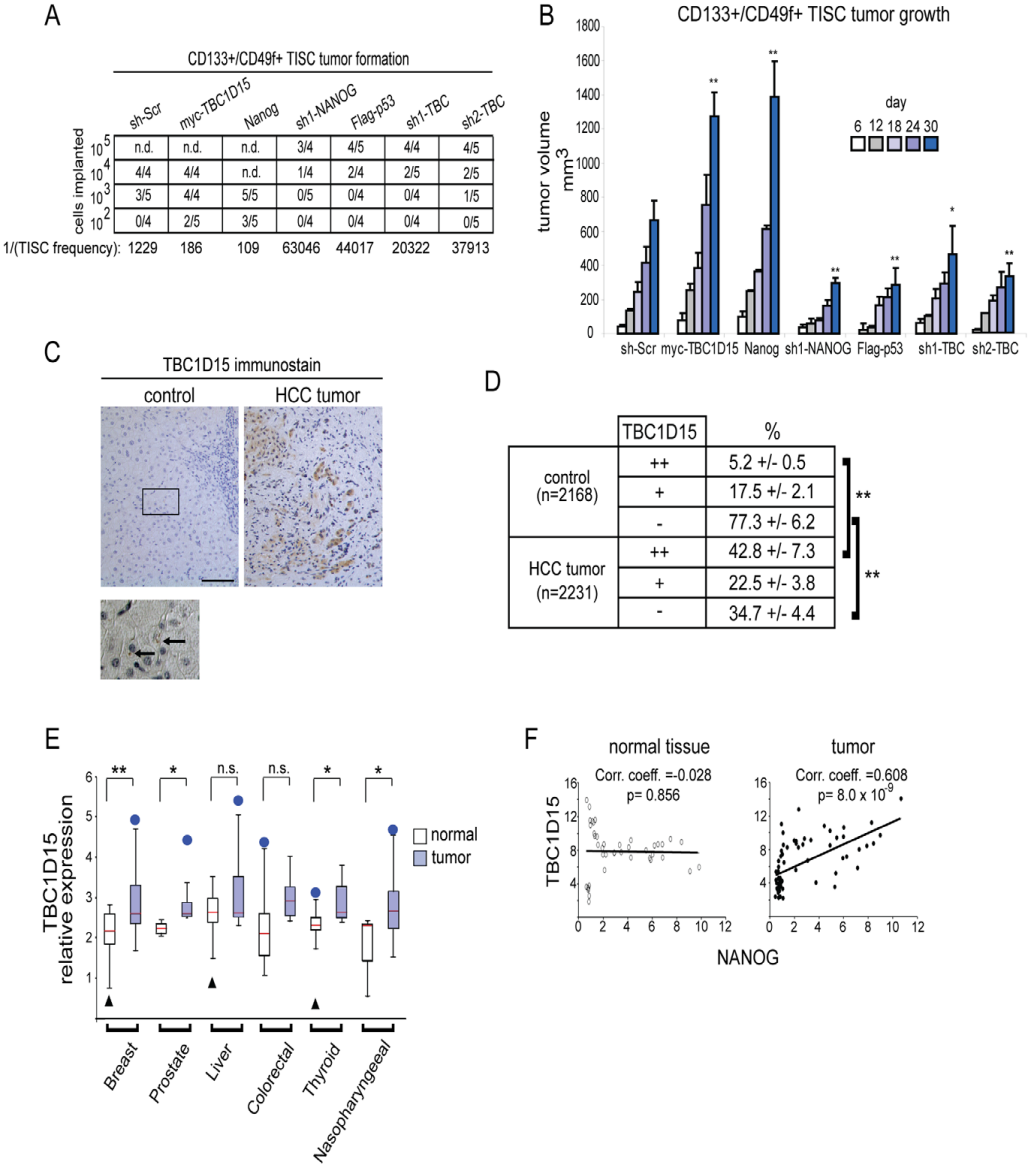
Oncogenic function of TBC1D15. (A) Tumor initiation titration. Defined numbers of TISCs stably expressing the indicated transgenes or lentivirus shRNAs were implanted subcutaneously into the dorsal hind flanks of NOG mice and tumor growth monitored for 60 days. Tumors greater than 25 mm^3^ and which exhibited growth progression during the course of the study were scored as positive. (B) Tumor growth kinetics. TISCs (5× 10^4^) were implanted subcutaneously into NOG mice as in (A) and tumor volumes were measured on the indicated days. At least 6 tumors were measured for each cell line examined. (C) TBC1D15 expression in HCC patient tissue specimens. Tumors and matched, non-cancerous control tissues were processed by sectioning and immunostained using TBC1D15 antibody, then counterstained with hematoxylin to indicate cell nuclei. Below, high magnification image showing punctate localization of TBC1D15 (arrows) in noncancerous control tissue. Scale bar, 50 μm. (D) Stained tissues from patient specimens were scored for the degree of TBC1D15 immunopositivity. Brackets indicate statistically significant (**P<0.01) comparisons between groups. The number of total cells scored across all samples (n = 17) is indicated for each group. (E) Box plot showing the expression of TBC1D15 in diverse tumor types and matched normal tissues. Red line indicates the median, box edges the 25–75 percentiles. Whiskers represent 1.5×interquartile range (IQR) above the third quartile or below the first quartile. Outliers (blue circles or black triangles) are shown when outside this range. (F) Scatter plots showing results from meta-analysis comparing the expression levels of *TBC1D15* and *NANOG* across multiple tumor samples and in matched, non-tumor control tissues.

To explore the significance of TBC1D15 in TISC-mediated oncogenesis, we carried out in vivo tumor formation titration assays in which defined numbers of TISCs were implanted subcutaneously into immune-compromised NOD/Shi-scid, *IL*-2*Rγ* null (NOG) mice. Stable expression of myc-TBC1D15 or Nanog enabled as few as 100 TISCs to form palpable tumors, while enforced expression of Flag-p53, or depletion of *TBC1D15,* impaired the efficiency of tumor formation (Figure 5A). The kinetics of tumor growth from these TISC lines also corresponded closely with their tumor formation efficiencies (Figure 5B). Furthermore, in surgically recovered tumor implants derived from TISCs expressing myc-TBC1D15, expression levels of the pluripotency markers *OCT4, SOX2* and *NANOG* were increased elevated relative to those measured in tumors expressing non-targeting shRNAs, Flag-p53 or an shRNA to deplete *TBC1D1*5 (Figures S6 and S7). In contrast, the expression *ALBUMIN,* a marker of differentiated hepatocytes, exhibited a reciprocal expression profile (Figure S7).

To further investigate a potential role for TBC1D15 in oncogenesis, we examined its expression in hepatocellular carcinoma (HCC) and in matched, adjacent noncancerous tissues obtained from surgically resected clinical specimens. A clinico-pathological description of these samples is provided in Table S1.Immunohistochemical analysis revealed a striking increase in TBC1D15 expression in HCC (Figure 5C). In noncancerous tissues, TBC1D15 immunostaining was detected in punctate, vesicular structures (Figure 5C), in line with its proposed function in endosomal and autophagosomal vesicle trafficking [29]. Quantitative scoring of stained specimens revealed that 42.8+/−7.3% of tumor cells, but only 5.2+/−0.5% of noncancerous control cells, were strongly immunopositive for TBC1D15 (*P*<0.01) (Figure 5D).

To address the generality of these findings, we carried out an extensive meta-analysis of *TBC1D15* expression in a panel of tumors arising from diverse tissue types and in matching, non-tumor control tissues. Through this approach, we detected significant (*P*<0.05) increases in *TBC1D15* expression in several tumor types, including those derived from breast, prostate, thyroid and nasopharyngeal tissues (Figure 5E). Additional studies are needed to address whether this observation extends to the expression of TBC1D15 at the protein level, which we observe in clinical HCC specimens, despite the lack of significant transcriptional induction as indicated by the meta-analysis. We also identified a correspondence in tumors between the expression of *TBC1D15* and *NANOG* (Figure 5F), in line with the analysis of liver TISC lysates (Figure 4A) and supporting a role for TBC1D15 in promoting TISC-mediated oncogenesis.

## Discussion

The molecular mechanisms underpinning the unchecked expansion of highly malignant tumor-initiating stem-like cells are not well understood. Here, we identify TBC1D15 as an oncoprotein that can competitively disengage the p53 tumor suppressor from its protective association with Numb, leading to proteolysis of p53 and to the deregulated propagation of tumor stem cell populations. Upon acute nutrient deprivation, TBC1D15 is subjected to autophagic degradation, thereby linking cellular energy and nutrient status to self-renewal capacity.

Our findings indicate that the Numb-p53 complex can be disengaged by TBC1D15. Interestingly, the TCTP oncoprotein was found in association with the Numb-p53 complex and shown to stimulate MDM2-mediated proteolysis of p53 [41]. These findings along with the data presented in this report collectively suggest that the Numb-p53 complex may serve as a pivotal control platform that integrates diverse inputs to enable the rapid modulation of cellular p53 levels. As we find no significant primary sequence homology between TCTP and TBC1D15, these proteins may dock with distinct subunits or epitopes within the Numb-p53 complex.

**Figure 6:**
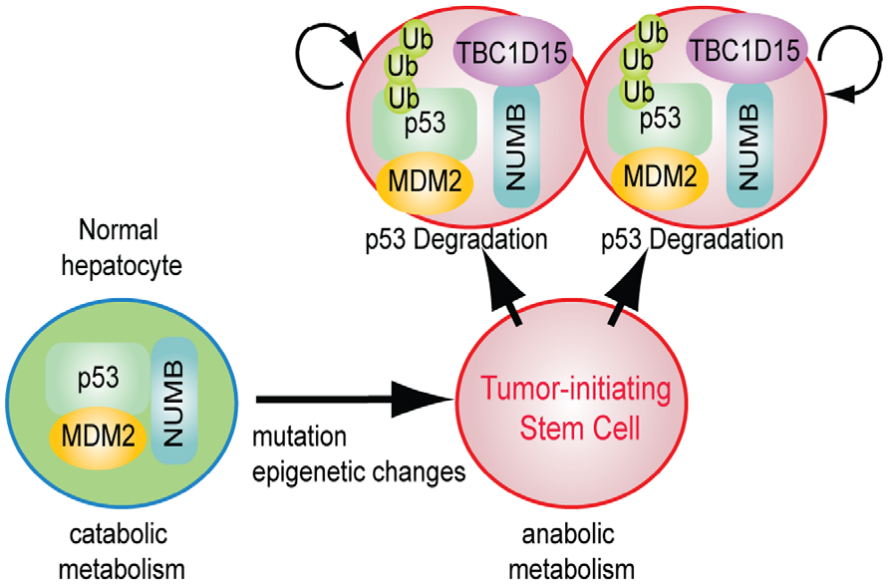
Conceptual model of TBC1D15 function in TISC-mediated oncogenesis. The transition from an untransformed hepatocyte to a TISC is accompanied by an anabolic shift and increased levels of the TBC1D15 oncoprotein, which destabilizes the Numb-p53 complex to promote deregulated self-renewal and oncogenesis.

The reciprocal and competitive destabilization between p53 and TBC1D15 provides a mechanism through which the cell may adopt distinct, self-reinforcing autophagy states. As contemplated by this proposal, under conditions where p53 activity is predominant, catabolism is favored and autophagic flux elevated [42], [43], leading to degradation of TBC1D15. Stabilization of p53 by components of the Vps34/Beclin complex through activation of the p53-debiquitinases USP10 and USP13 [44] may compound this effect. Direct repression of *Tbc1d15* through association of p53 with promoter-proximal sequences may also occur and is consistent with data obtained from genome-wide identification of p53 binding sites [19]. On the other hand, when TBC1D15 expression is elevated, p53 levels are diminished and the rate of autophagosome delivery to the lysosome is attenuated [30], stabilizing TBC1D15 and effectively entrenching the hypoautophagic state. This effect may be reinforced through the accumulation of p62, an upstream activator of the mTORC1 anabolic kinase complex, upon attenuated autophagic flux [45], [46]. Conditions that alter the competitive balance in favor of TBC1D15, such as its profound overexpression in tumors, may reverse the virtuous, p53-driven cycle into a vicious one that supports deregulated self-renewal in TISCs. This conceptual model is depicted schematically in Figure 6.

Our findings indicate an inverse relationship between self-renewal and catabolism and suggest that a threshold level of anabolic metabolism must be maintained in pluripotent stem cells. Such a proposal is consistent with a shift away from mitochondrial respiration and towards glycolysis in pluripotent cells [47]–[49] and in oncogenically transformed cells [50], [51], and resonates with the demonstration that master anabolic regulator mTORC1 supports tumorigenesis and the long-term self-renewal of pluripotent stem cells [52]. The functional relationship between pluripotency and oncogenesis is underscored by the finding that the core pluripotency-associated TFs, including c-Myc, Oct4, Sox2, Lin-28 and Nanog, promote oncogenic transformation and are recurrently overexpressed or amplified in human malignancies [7], [9]–[11], [53]. Through its inhibition of p53 and potential modulation of additional downstream effectors [29], [30], TBC1D15 may act to reconfigure cell metabolism and promote the pluripotent state and oncogenic transformation. While TBC1D15 supports self-renewal in TISCs and reprogramming efficiency in cultured NGFP2 MEFs (Figure 4), further studies into its role in the acquisition and maintenance of pluripotency, and to address whether TBC1D15-deficient ESCs can develop into the full range of adult tissues, are warranted.

## Materials and Methods

### Mouse Strains

HCV Ns5a-Tg mice have been described previously [23]. Mice harboring a conditionally deleted *Atg5 ^fl/fl^* allele [54], [55] and intercrossed with *Alb-cre* mice were generously provided by Dr. James Ou (University of Southern California, Keck School of Medicine). Homozygous *Trp53^−/−^* mice (B6.129S2-Trp53^tm1Tyj^/J) were obtained from Jackson Laboratory (Stock Number 02101). NOD/Shi-scid, *IL*-2*R*γ null (NOG) mice were obtained from the Central Institute for Experimental Animals (Kanagawa, Japan).

All mice were raised in a specific pathogen-free environment at the University of Southern California Keck School of Medicine. All animal procedures described herein and performed specifically for this study were approved by the USC Keck Medical Center Institutional Animal Care and Use Committee (IACUC) protocols 11025 and 11403. All surgeries were performed under deep anesthesia, and all efforts were made to minimize suffering.

### Cells and Reagents

Murine TISCs were isolated from liver tumors resected surgically prior to euthanasia from NS5a-Tg mice [56] maintained for 12 months on a Lieber-Decarli diet containing 3.5% alcohol [23]. CD133+/CD49f+ murine hepatoma TISCs were isolated using the anti-Prominin-1 (CD133) MACS affinity column (Miltenyi Biotech) following mechanical dissociation of liver tumors in sterile PBS and digestion with 1 mg/mL collagenase/dispase solution (Roche) for 45 min at 37°C. The unbound column flow-through fraction (CD133-) was also recovered for propagation. Both fractions were maintained in TISC culture medium [57]. PIL-4 hepatoblasts (p53-null) were generously provided by Dr. Aleksandra Filipovska (University of Western Australia, Crawley, Australia).The NGFP2 iPS MEFs (Stemgent) contain a constitutively expressed reverse tetracycline transactivator (rtTA) driven by the ROSA26 promoter (R26-M2rtTA) and GFP knocked in to the endogenous *Nanog* locus (Nanog-GFP) as well as doxycycline-inducible transcription factors Oct4, Sox2, Klf4, and c-Myc. NGFP2 MEFs were seeded in gelatin-coated, 12-well plates and cultured in MEF culture medium (DMEM, 15% FBS, 0.1 mM non-essential amino acids (NEAA), 1 mM sodium pyruvate, 50 μg/mL each penicillin and streptomycin, 0.1 mM β-mercaptoethanol). Two days after the addition of doxycycline to the culture media, the media was replaced with reprogramming medium (Knockout DMEM (Invitrogen), 10% knockout serum replacement (Invitrogen), 5% FBS, 1 mM sodium pyruvate, 0.1 mM β-mercaptoethanol).

The following chemical reagents were purchased from Sigma: antimycin A (A8674), Bafilomycin A1 (B1793), etoposide (E1383), FCCP (C2920), lactacystin (L6785), Nutlin-3 (N6287), oligomycin (75351), PP242 (P0037), rapamycin (R0395) and rotenone (R8875).

The following commercial antibodies were used: ATG5 (Santa Cruz Biotechnology, sc-33210), Flag (Sigma, F1804), GFP (Santa Cruz Biotechnology, sc-20088×), c-Myc (Abcam, 9E11), Nanog (Abcam, ab80892), Numb (H-70, Santa Cruz Biotechnology, sc-25668), p53 (Santa Cruz Biotechnology, sc-17846), p70 S6 kinase (Cell Signaling Technology, 49D7), phospho-p70 S6 kinase (T389) (Cell Signaling Technology, 1A5) and actin (Sigma, A5316).

To generate the TBC1D15 antibody, a rabbit polyclonal antibody was raised by Syd Labs (Malden, MA) against a conserved peptide sequence (KELPQAVCEILGLQ) near the carboxyl-terminus of TBC1D15, followed by two sequential rounds of affinity purification.

### Vectors

The pBabe.puro myc-TBC1D15 vector encoding murine TBC1D15 was generously provided by Dr. Aimee Edinger (University of California, Irvine). CA-aPKCζ.Flag [58] was obtained from Addgene (Plasmid 10802). The pMXs-p53 and pMXs-p53D278N expression vectors [59] were obtained from Addgene (Plasmids 22725 and 22727, respectively). The Flag-p53 plasmid, encoding human p53 containing an amino-terminal Flag tag [60], was obtained from Addgene (Plasmid 10838). The vectors for pcDNA3-Beclin [61] and pET15-human p53 [62] were obtained from Addgene (Plasmids 21150 and 24859, respectively). To generate the Flag-TBC1D15 expression constructs, TBC1D15 DNA segments containing NotI and BglII restriction sites were generated by PCR using Pfu polymerase and myc-TBC1D15 as a template. Products were subcloned into the p3xFlag-CMV-7.1 vector (Sigma). To generate the EGFP-TBC1D15 fusions, TBC1D15 PCR products containing EcoRI and SalI sites were subcloned into the pEGFP-C2 vector (Takara Bio).

Lentiviral shRNAs in pTRC1 or pTRC2 vector backbones were purchased from Sigma. The following lentivirus clones and sequences were used for depletion of murine (Mm) or human (Hs) RNAs:

Hs TBC1D15, sh1: TRCN0000154685, 5′-CCGGGAGGTAATGTGGACCGAACTACTCGAGTAGTTCGGTCCACATTACCTCTTTTTTG-3′;

Hs TBC1D15, sh2: TRCN0000231963, 5′-CCGGGCATTAGATTCCTCTAGTATTCTCGAGAATACTAGAGGAATCTAATGCTTTTTG-3′.

Mm TBC1D15, sh1: TRCN0000250021, 5′-CCGGTGATTCTGCTTCACGACATTTCTCGAGAAATGTCGTGAAGCAGAATCATTTTTG-3′;

Mm TBC1D15, sh2: TRCN0000250023 5′-CCGGTTAACACCTGCATGATCATTTCTCGAGAAATGATCATGCAGGTGTTAATTTTTG-3′.

Mm Nanog, sh1: TRCN0000075333,

5′-CCGGGCCAACCTGTACTATGTTTAACTCGAGTTAAACATAGTACAGGTTGGCTTTTTG-3′,

Mm Nanog, sh2: TRCN0000075334,

5′-CCGGGCCAGTGATTTGGAGGTGAATCTCGAGATTCACCTCCAAATCACTGGCTTTTTG-3′.

The sh-Scr control lentivirus was obtained from Addgene (Plasmid 1864) [63] and expresses a hairpin of the following sequence: 5′-CCTAAGGTTAAGTCGCCCTCGCTCGAGCGAGGGCGACTTAACCTTAGG-3′.

### Transfection and Lentivirus Transduction

Cultured cells at 60–70% confluence were transfected with plasmid vector using the Bio-T reagent (Bioland Scientific, Paramount, CA) according to the manufacturer’s protocol. Lentivirus particles were generated by co-transfecting subconfluent HEK-293T cells with lentivirus vector along with the psPAX2 packaging vector and an envelope vector, pMD2.G. Virus was collected from the culture media at 48 and 72 hr post-transfection and purified by ultracentrifugation in a Beckman SW28 rotor (20,000 rpm, 2 hr). Viral pellets were resuspended in 1× PBS and stored at −80°C until use. Cells were transduced with virus at an MOI of 0.5–0.6 in the presence of polybrene (5 μg/mL). Transduced cells were selected in culture media containing puromycin (2 μg/mL).

### Affinity Purification of Numb Complexes

Early-passage, subconfluent CD133+/CD49f+ murine TISCs (2×10^8^) cultured in 500 cm^2^ plates were washed in PBS and lysed using the NE-PER protein extraction kit (Thermo Fisher) supplemented with Complete Mini Protease Inhibitor Cocktail (Roche). Aliquots of cytoplasmic lysate (300 μL) were loaded onto continuous 5–40% sucrose gradients (4.75 mL) and resolved by ultracentrifugation in a Beckman SW55Ti rotor for 2.5 hr at 50,000 rpm (∼170,000×g). Fractions were collected, pooled and pre-cleared with protein G-PLUS agarose beads (200 μL) (Santa Cruz Biotechnology) for 1 hr at 4°C, then incubated overnight with either anti-GFP or anti-Numb (H-70) antibody (Santa Cruz Biotechnology). A 25% slurry of Protein G-PLUS agarose (100 μL) was added for 2.5 hr at 4°C with gentle rotation, and pellets were washed three times with Buffer W (20 mM Tris pH 7.3, 300 mM NaCl, 0.5% triton X-100, 2% glycerol), twice with PBS, and once with 0.1X PBS. Bound proteins were incubated twice with 70 μl elution buffer (10 mM ammonium carbonate, 8 M urea) for 50 min, lyophilized in a speed vacuum, and frozen at −20°C until analysis by liquid chromatography and tandem mass spectrometry.

### Mass Spectrometry and Proteomics Analysis

Peptides were processed as described previously [26]. In brief, lyophilized proteins recovered from immunoprecipitates were resuspended in 50 mM ammonium bicarbonate (pH 8.0) with 5% acetonitrile and sequencing grade trypsin (Promega) at a concentration of 12.5 ng/mL and incubated at 37°C for 8 hours. Trypsin reactions were quenched by addition of 5% formic acid and tryptic peptides were loaded onto the LTQ high performance linear ion trap mass spectrometer, which was controlled by Xcalibur software. Each scan was set to acquire a full MS scan, followed by MS/MS scans on the four most intense ions from the preceding MS scan. Relative collision energy for collision-induced dissociation was set at 35%. At least two replicate runs were performed for each sample, and all peptides identified in separate runs were pooled into one list for further analysis.

### Cell Lysis and Immunoprecipitation

Cells cultivated in 10 cm plates were washed in PBS, gently lifted from the plates and lysed using the NE-PER protein extraction kit supplemented with Complete Mini Protease Inhibitor Cocktail. Aliquots of cytoplasmic lysate (300–500 μL) were incubated with the appropriate antibody (5–7 μg) for 6 hr at 4°C. A 25% slurry of Protein G-PLUS agarose (40 μL) was added for an additional 2.5 hr at 4°C with gentle rotation, and pellets were washed five times with Buffer W (20 mM Tris pH 7.3, 300 mM NaCl, 0.5% triton X-100, 2% glycerol). Samples were processed by boiling in 5× Laemmle sample buffer prior to analysis by SDS-PAGE.

### Protein Identification and Quantification

A database of semitryptic peptides, generated from the NCBI mouse database, was imported into Sorcerer 2 (SageN Research). The MS/MS spectra obtained from MudPIT were automatically extracted and searched against the database using Sorcerer SEQUEST. For the search, 57 Da was added to all cysteines to account for carboxyamidomethylation. An additional 42 Da was permitted on N-terminal residues to account for potential acetylation, and 16 Da was permitted on methionines to account for potential oxidation. The results from the SEQUEST searches were automatically filtered, organized, and displayed by PeptideProphet and ProteinProphet (Institute for Systems Biology). ProteinProphet computed a probability score from 0 to 1 for each protein, based on peptides assigned to MS/MS spectra and analyzed by.

PeptideProphet. To minimize false positive identification, the thresholds for adjusted probability score for each peptide and for protein identification were set at g = 0.85. The false positive rate using these criteria was below 1%, based on decoy database analysis.

### Purification of Recombinant Proteins

To purify His_6_-p53, BL21 (DE3) Rosetta-gami 2 *E. coli* (EMD-Millipore) harboring the pET15-p53 vector were cultured at 37°C until reaching A600 = 0.6, then induced with IPTG (0.5 mM) and grown for an additional 3 hr at 30°C. Bacterial pellets were resuspended in ice-cold bacterial lysis buffer (PBS, 0.5% triton X-100, 1 mM DTT, 0.2 mg/mL lysozyme) for 30 min, then sonicated five times in 30-s pulses with 1 min on ice between pulses. Following a centrifugation (10,000×g, 5 min) the supernatant was recovered and incubated with Ni-NTA agarose resin (Qiagen) for 5 hr with tumbling. Pellets were recovered by gentle centrifugation (1300×g, 5 min), washed three times in lysis buffer containing 5 mM imidazole, and eluted with elution buffer (PBS, 200 mM imidazole, 5% glycerol). Samples were subsequently dialyzed against PBS containing 5% glycerol and 1 mM DTT prior to storage at −80°C.

To purify myc-TBC1D15 and Flag-Numb variants, HEK-293A cells cultured in 500 cm^2^ plates and grown to 70–80% confluence were transfected with either myc-TBC1D15, Flag-Numb-3A or Flag-Numb-3D. 48 hr post-transfection, cells were washed once in cold PBS, gently lifted from the plate, and lysed in 3 mL cold Lysis Buffer (10 mM Hepes pH 7.5, 10 mM KCl, 1.5 mM MgCl_2_, 0.5% NP-40) for 1 hr on ice, followed by centrifugation at 13,000 × g for 5 min and recovery of the supernatant. Immunoprecipitations were performed by incubating 1.5 mL supernatant with 80 μl of a 40% slurry of M2 anti-Flag agarose or with 6 μg anti-myc antibody for 2 hr at 4°C with tumbling. For anti-myc immunoprecipitations, 80 μl of a 25% slurry of protein G-agarose was added, and all samples were incubated an additional 2 hr with tumbling. Beads were washed once in Buffer W (20 mM Tris pH 7.3, 300 mM NaCl, 0.5% triton X-100, 2% glycerol), once in PBS containing 0.2% Triton X-100, and once in PBS. Flag-Numb variants were eluted by incubation in 3×Flag peptide (1 mg/mL) overnight at 4°C. All samples were stored at −80°C in PBS containing 5% glycerol and 1 mM DTT.

### In vitro Protein Interaction Assays

Purified recombinant Flag-Numb-3A or Flag-Numb-3D (1 μg) were incubated with myc-TBC1D15-coated agarose resin (∼ 1 μg myc-TBC1D15) in 400 μl total volume for 45 min at room temperature with tumbling. Samples were washed twice in Buffer W and once in PBS, then boiled in 5× Laemmle sample buffer prior to analysis by SDS-PAGE. For competition assays, recombinant His_6_-p53 (1.0–20 nM) was added to the binding reaction prior to addition of either agarose resin or anti-Numb agarose.

### Propidium Iodide Staining

HEK-293A cells were treated for 16 hr with DMSO vehicle or with etoposide (34 μM), harvested by trypsinization and fixed for 25 min at −20°C in PBS containing 80% ethanol. Cells were pelleted and resuspended in PBS containing 50 μg/mL propidium iodide and 0.1 mg/mL RNase A (Qiagen) and incubated for 40 min at 37°C. Cells were washed twice with PBS prior to FACS analysis.

### Flow Cytometry Analysis

NGFP2 cells cultured in the absence or presence of doxycycline were trypsinized, washed twice with PBS, and resuspended in FACS buffer (PBS +5% goat serum) prior to analysis on a Beckman Coulter Cyan ADP Analyzer. Results were processed using the Cytomation Summit software suite.

### Colony Formation Assay

Murine TISC lines were trypsinized and seeded in triplicate 12-well plates (5×10^4^ cells/well). Cells were plated in methylcellulose media (Z403, Stemgent) containing 30% TISC culture medium (DMEM-F12, 10% FBS, 100 nM dexamethasone, 1× nucleosides (Sigma) and 20 nM murine EGF) and allowed to grow for 1 week. Cells were harvested by trypsinization and viable, trypan blue-excluding cells were counted using an automated cell counter (Invitrogen) followed by replating. Colonies containing greater than 25 cells were scored as positive.

### Metabolic Flux Analysis

Murine TISCs (5 × 10^4^) were plated in gelatin-coated 24-well cell culture microplates compatible for use in the Seahorse XF-24 extracellular flux analyzer (Seahorse Bioscience, North Billerica, MA). The following day, cells were incubated in pre-warmed serum-free DMEM medium containing 2 mM GlutaMAX, 1 mM sodium pyruvate, and 25 mM glucose for 1 hr. The oxygen consumption rate (OCR) and extracellular acidification rate (ECAR) were measured by the XF-24 extracellular flux analyzer at basal levels and following sequential exposure to oligomycin (2 μM), FCCP (carbonyl cyanide-*p*-trifluoromethoxyphenylhydrazone) (0.5 μM) and a mix of antimycin A (5 μM )and rotenone (1 μM). The OCR and ECAR were determined by plotting the oxygen tension or acidification of the medium in the chamber, respectively, and normalized by protein concentration (pmol/min/mg). At least three biological replicates were performed for each analysis.

### Tumor Implants

NOG mice were purchased from Jackson Laboratories and housed under pathogen-free conditions. Mice were provided with normal chow ad libitum. Liver TISCs (5×10^4^) were resuspended in 100 μL of 50% Matrigel (BD Biosciences) in PBS and injected subcutaneously into the dorsal hind flanks of anesthetized mice. Tumors were measured with calipers and the volume calculated according to the formula V = a×b^2^/2, where a represents the largest and b the smallest superficial diameters [64].

### Tumor Lysate Preparation

Tumors were surgically removed from deeply anesthetized mice, then mechanically dissociated and dounce homogenized in ice cold RIPA buffer (50 mM Tris HCl pH 7.4, 150 mM NaCl, 2 mM EDTA, 1% NP-40, 0.1% SDS) supplemented 10 mM NaF, 1 mM Na_3_VO_4_ and 1 tablet of protease inhibitor cocktail (Roche) per 10 mL buffer. Lysates were centrifuged (13,000 x g, 10 min) and supernatants recovered for SDS-PAGE and immunoblotting analysis.

### Quantitative Reverse-transcription PCR

Total RNA was prepared from mechanically dissociated tumor tissue using TRIzol (Invitrogen) and purified using the RNeasy mini kit (QIAGEN) with DNase I treatment. First-strand cDNA was synthesized with SuperScript III reverse transcriptase (Invitrogen) at 48°C for 40 min before PCR cycling. Quantitative PCR was performed with 2× SYBR Green Master Mix (Applied Biosystems). All reactions were run on an ABI 7300 HT Real-Time PCR instrument with a 5-min hot start at 95°C followed by 40 cycles of a three-step thermocycling program: 15 s denaturing at 94°C, 15 s annealing at 55°C, and a 20-s extension at 70°C. Gene expression was determined relative to GAPDH control via the ΔCt method. The following forward and reverse primers, respectively, were used for PCR analysis of RNA transcripts:

OCT4: (5′-GGCGAGGCCTTTCCCTCTGT-3′) and (5′-CTCAGTAAAAGAATTTAACC-3′).

SOX2: (5′-TGCGAACTGGAGAAGGGGAGAG-3′) and (5′-CGCAGCTGTCGTTTCGCTGCGG-3′).

NANOG: (5′-GCAGCAAAACTTCTCTGCCA-3′) and (5′-GTAAGTCTCATATTTCACCT-3′).

ALBUMIN: (5′-CATGACACCATGCCTGCTGAT-3′) and (5′-GCCTTTCCACCAGGGATCCAC-3′).

TIGAR (5′-GATGAGTCTCTTTATCATAA-3′) and (5′-TGGTTAGATTTTGATGCAG-3′).

SCO2 (5′-TCTACCTGCTCAACCCTGAC-3′) and (5′-CGTTTAATGATGGGGCCCAG-3′).

SESN2 (5′-CTGTACGCCCTCCGTGCCAT-3′) and (5′-GCGTGGGGAGGGTGGGCATG-3′).

DRAM (5′-CACCCTAAGGATATCCACAG-3′) and (5′-TC CCAATAATCAGGGTGGC-3).

GAPDH: (5′-TGTGTCCGTCGTGGATCTGA-3′) and (5′-AAGTTGCAGGAGACAACCTGGTC-3′).

### Human Tissue Specimens

Sections from formalin-fixed and paraffin-embedded human hepatocellular carcinoma (HCC) and matched normal liver tissue surgical specimens were obtained from the University of Minnesota Liver Tissue Cell Distribution System. All tissues were collected with informed patient consent, in writing, that was granted prior to surgery in accordance with a protocol approved by the Institutional Review Board at the University of Minnesota, Minneapolis. In all, seventeen sets of HCC tumor and matched normal tissue samples (10 M, 7 F) were obtained from deidentified patients (43–67 years of age) with alcohol- and/or viral hepatitis-associated liver tumors of varying grades. Non-tumor control tissues were obtained from clearly delineated margins of surgical specimens.

### Immunostaining

Tissue sections were rehydrated by immersion in xylene (3×5 min) and 100% ethanol (2×5 min) followed by a series of hydrated ethanol baths and PBS. To enhance antigen recovery, rehydrated sections were immersed in 10 mM sodium citrate (pH 6.0) at 90°C for 15 min and washed twice in PBS. All tissue sections were blocked in blocking buffer (PBS, 5% fetal goat serum) and probed with primary antibodies at a dilution of 1∶400 in blocking buffer, followed by washes (3×5 min) in PBS and incubation with HRP-conjugated secondary antibody (1∶500). Staining was visualized using the DAB Histochemistry kit (Invitrogen) and analyzed by blinded scoring of cell staining intensity.

### Statistical Analysis

Statistical significance was estimated by unpaired, two-tailed Student’s t test. Bars represent the mean and error bars the SD. For all Figures, statistical significance is represented by asterisks above each column: *P<0.05; **P<0.01. TISC frequency was calculated from tumor formation titration experiments using the limdil function of the statmod package in the R-statistical software suite.

For gene expression meta-analysis, microarray expression data corresponding to patient samples from Breast (GSE 9574, GSE2429); Prostate (GSE3325); Liver (GSE19665); Colorectal (GSE15960); Thyroid (GSE3678) and Nasopharyngeal (GSE13597) cancers along with matching, non-tumor control tissues were obtained from the Gene Expression Omnibus (http://www.ncbi.nlm.nih.gov/geo). The signal intensities were extracted using the PLIER 16 (probe logarithmic intensity error) algorithm and data was quantile normalized. The correlation of expression between TBC1D15 and NANOG was calculated using Pearson’s correlation coefficient and R-statistical software.

## Supporting Information

Figure S1 **Interaction of endogenous Numb and TBC1D15 in diverse cell types.** Lysates prepared from PIL-4 hepatoblasts (A) or HeLa cervical carcinoma cells (B) were subjected to immunoprecipitation using TBC1D15 antibody or anti-Numb agarose resin. Immunoprecipitates and cytoplasmic lysates corresponding to 10% of the input volume used in the immunoprecipitation were analyzed by SDS-PAGE followed by immunoblotting using the indicated antibodies.
(TIF)

Figure S2 **Purification of recombinant proteins.** Coomassie brilliant blue-stained SDS-PAGE gel showing efficient purification of recombinant GST, Flag-Numb-3A, Flag-Numb-3D as well as agarose resin coated with myc-TBC1D15 isolated from HEK-293A cell lysates. Recombinant His_6_-p53 was purified from bacterial lysates.
(TIF)

Figure S3 **Domain structure and sequence homology of TBC1D15 and Canoe.** Schematic diagram showing the major domains of murine TBC1D15 and *Drosophila* Canoe. Polypeptide sequence conservation within the region of homology is shown below. RA, Ras-association domain; FHA, forkhead domain; PDZ, PSD-95, Dlg, and ZO-1 domain; F-actin, actin-binding domain.
(TIF)

Figure S4 **TBC1D15 2KR mutants are susceptible to p53-mediated antagonism.** Wild-type and 2KR mutant forms of Flag-TBC1D15 and Flag-TBC1D15-N were expressed with either GFP vector control or myc-p53, followed by lysis and immunoblotting using the indicated antibodies.
(TIF)

Figure S5 **Gene silencing by lentivirus shRNAs.** Lysates prepared from murine TISCs stably expressing the indicated lentivirus shRNAs were resolved by SDS-PAGE and analyzed by immunoblotting using the indicated antibodies to assess depletion efficiency.
(TIF)

Figure S6 **Effect of TBC1D15 on cellular metabolism.** (A) Lysates prepared from murine CD133+ TISCs expressing the indicated vectors or depleted for *TBC1D15* were resolved by SDS-PAGE and analyzed by immunoblotting for LC3. (B) Expression of the indicated genes was examined by qRT-PCR analysis of RNA transcripts isolated from TISCs stably expressing a control vector, sh1*-TBC1D15* or myc-TBC1D15. **P<0.01 relative to vector control. (C and D) The basal extracellular acidification rate (ECAR) (C) and oxygen consumption rate (OCR) (D) were determined using the Seahorse XF-24 metabolic flux analyzer for TISCs stably expressing either control vector or myc-TBC1D15 or following depletion of *TBC1D15*. The OCR was measured over time in approximately 7 min intervals. The first three measurements were conducted to establish a baseline rate, followed by three measurements after the addition of oligomycin, an ATPase inhibitor. Following uncoupling of the proton gradient with FCCP, the maximum OCR rates were determined over the next three time intervals. A final series of measurements were conducted after inhibition of the mitochondrial respiratory chain with antimycin and rotenone. All experiments were conducted in triplicate.
(TIF)

Figure S7 **Analysis of gene expression in resected tumor implants**. Implanted tumors derived from the indicated TISC lines were harvested surgically and mechanically dissociated, followed by extraction of total RNA. Expression levels of the indicated genes was determined by quantitative RT-PCR. At least three independent biological replicates were performed for each sample. Error bars represent the standard deviation.
(TIF)

Table S1 **Clinicopathologic features of patient tissue samples.** Clinicopathologic characteristics of patient HCC tumor and matched normal tissue samples that were included as part of this study and scored for TBC1D15 immunoreactivity. NASH, non-alcoholic steatohepatitis.
(TIF)
