# Supplementary figures and images for: The TBC1D15 Oncoprotein Controls Stem Cell Self-Renewal through Destabilization of the Numb-p53 Complex
Source: PLoS One. 2013 Feb 27;8(2):e57312. doi: 10.1371/journal.pone.0057312 (PMC3584131; doi:10.1371/journal.pone.0057312)

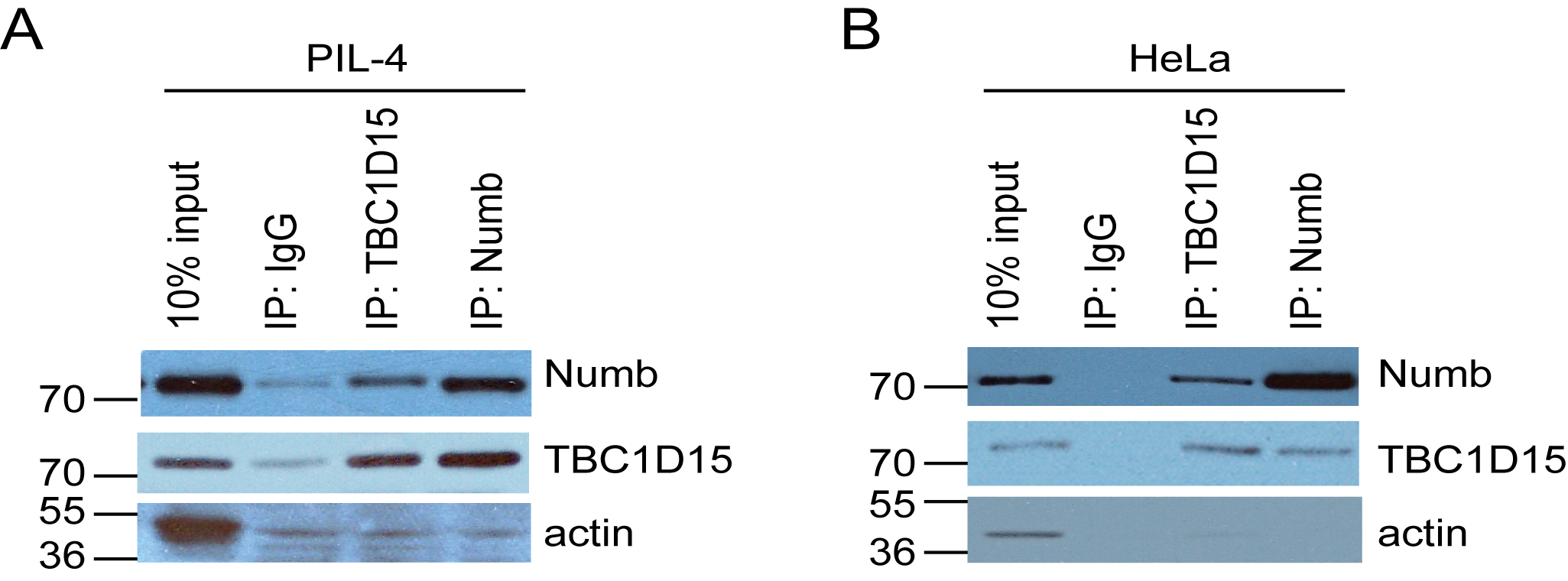

Supplement: Figure S1 — Interaction of endogenous Numb and TBC1D15 in diverse cell types. Lysates prepared from PIL-4 hepatoblasts (A) or HeLa cervical carcinoma cells (B) were subjected to immunoprecipitation using TBC1D15 antibody or anti-Numb agarose resin. Immunoprecipitates and cytoplasmic lysates corresponding to 10% of the input volume used in the immunoprecipitation were analyzed by SDS-PAGE followed by immunoblotting using the indicated antibodies. (TIF) [file pone.0057312.s001.tif]

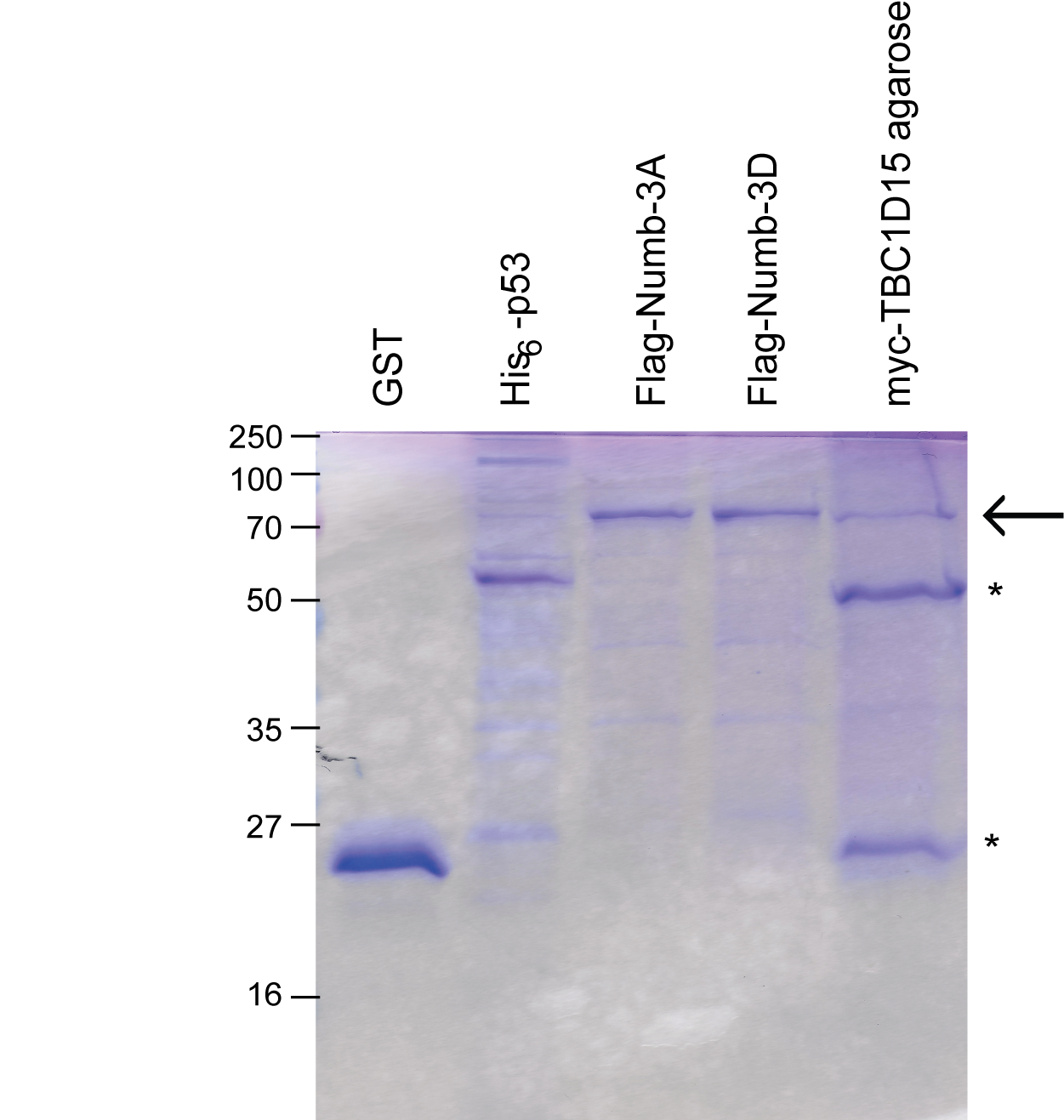

Supplement: Figure S2 — Purification of recombinant proteins. Coomassie brilliant blue-stained SDS-PAGE gel showing efficient purification of recombinant GST, Flag-Numb-3A, Flag-Numb-3D as well as agarose resin coated with myc-TBC1D15 isolated from HEK-293A cell lysates. Recombinant His6-p53 was purified from bacterial lysates. (TIF) [file pone.0057312.s002.tif]

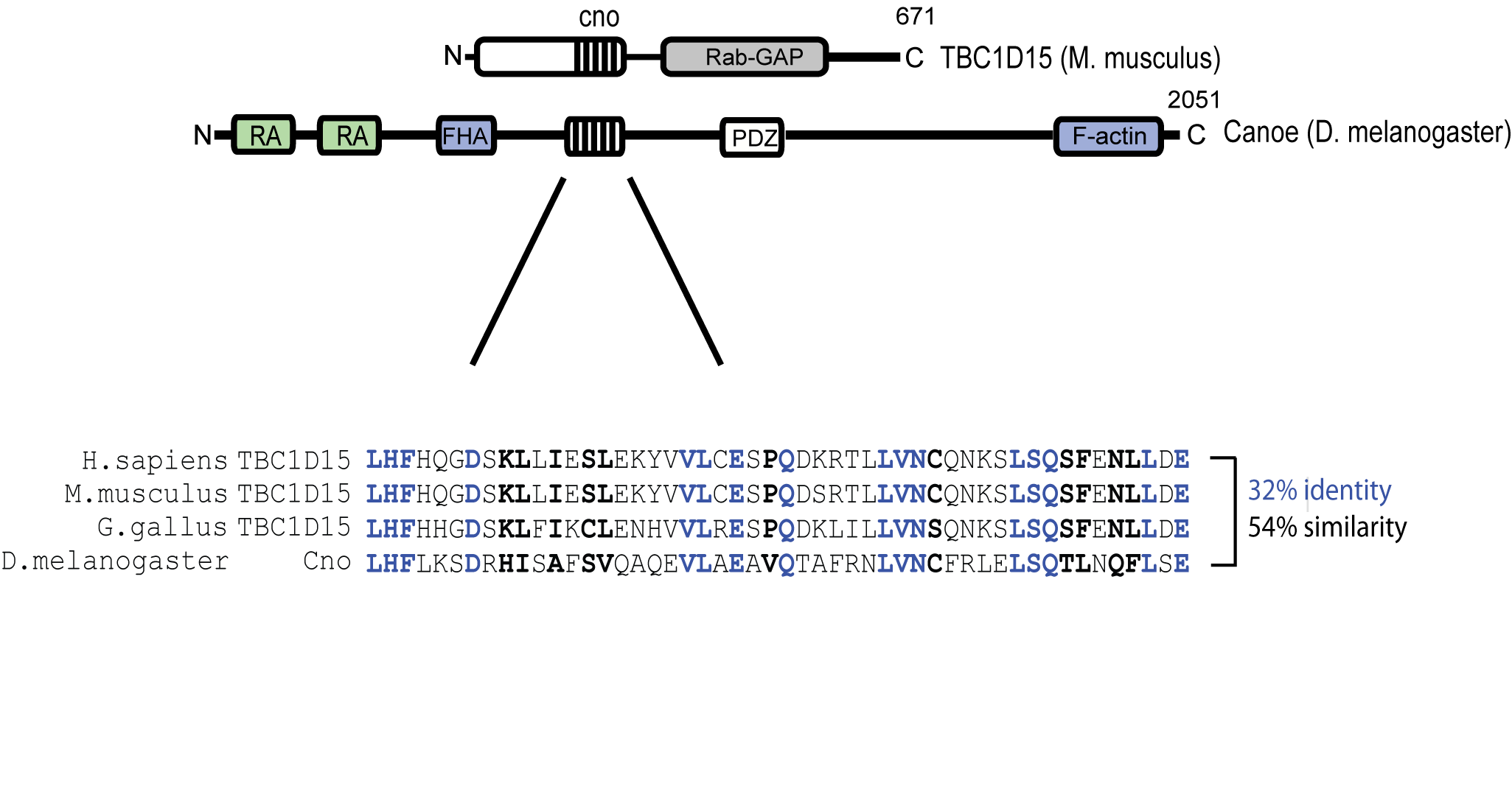

Supplement: Figure S3 — Domain structure and sequence homology of TBC1D15 and Canoe. Schematic diagram showing the major domains of murine TBC1D15 and Drosophila Canoe. Polypeptide sequence conservation within the region of homology is shown below. RA, Ras-association domain; FHA, forkhead domain; PDZ, PSD-95, Dlg, and ZO-1 domain; F-actin, actin-binding domain. (TIF) [file pone.0057312.s003.tif]

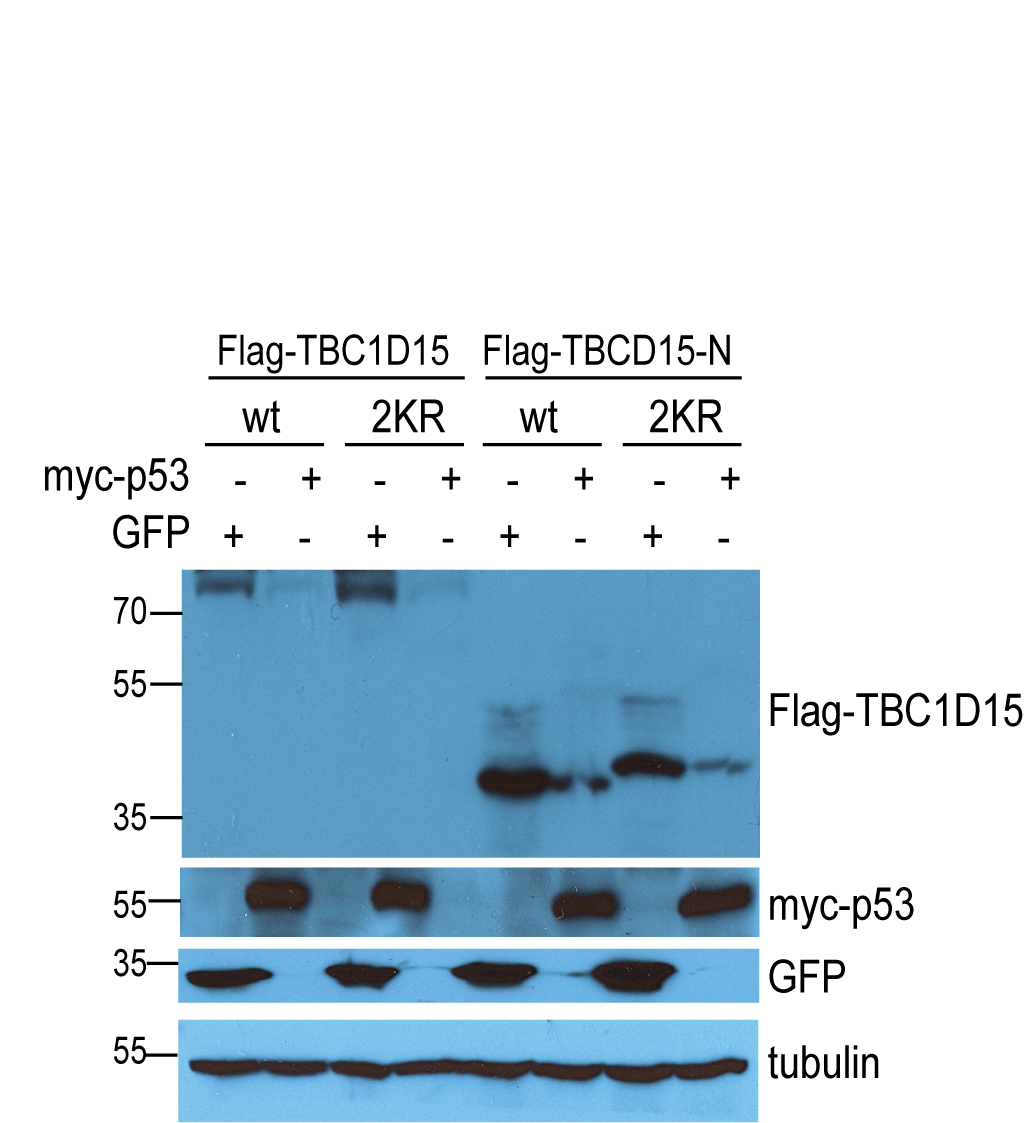

Supplement: Figure S4 — TBC1D15 2KR mutants are susceptible to p53-mediated antagonism. Wild-type and 2KR mutant forms of Flag-TBC1D15 and Flag-TBC1D15-N were expressed with either GFP vector control or myc-p53, followed by lysis and immunoblotting using the indicated antibodies. (TIF) [file pone.0057312.s004.tif]

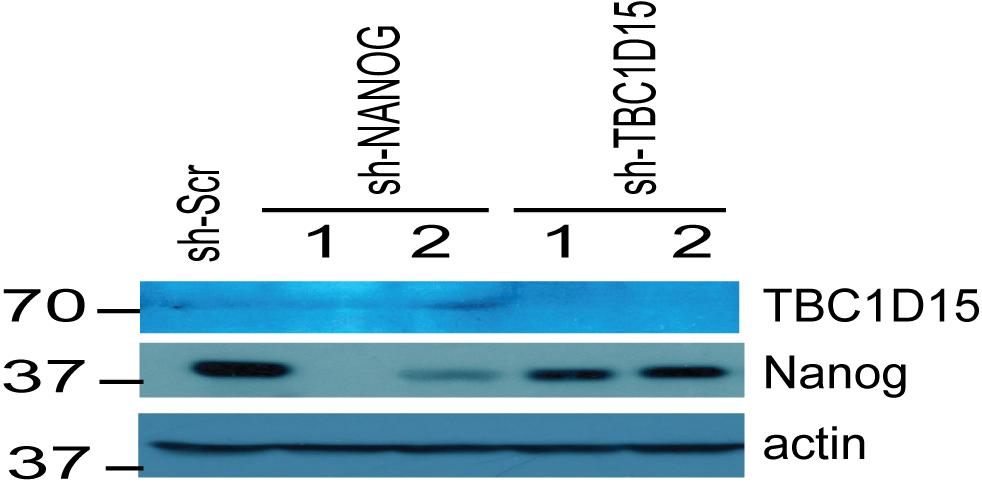

Supplement: Figure S5 — Gene silencing by lentivirus shRNAs. Lysates prepared from murine TISCs stably expressing the indicated lentivirus shRNAs were resolved by SDS-PAGE and analyzed by immunoblotting using the indicated antibodies to assess depletion efficiency. (TIF) [file pone.0057312.s005.tif]

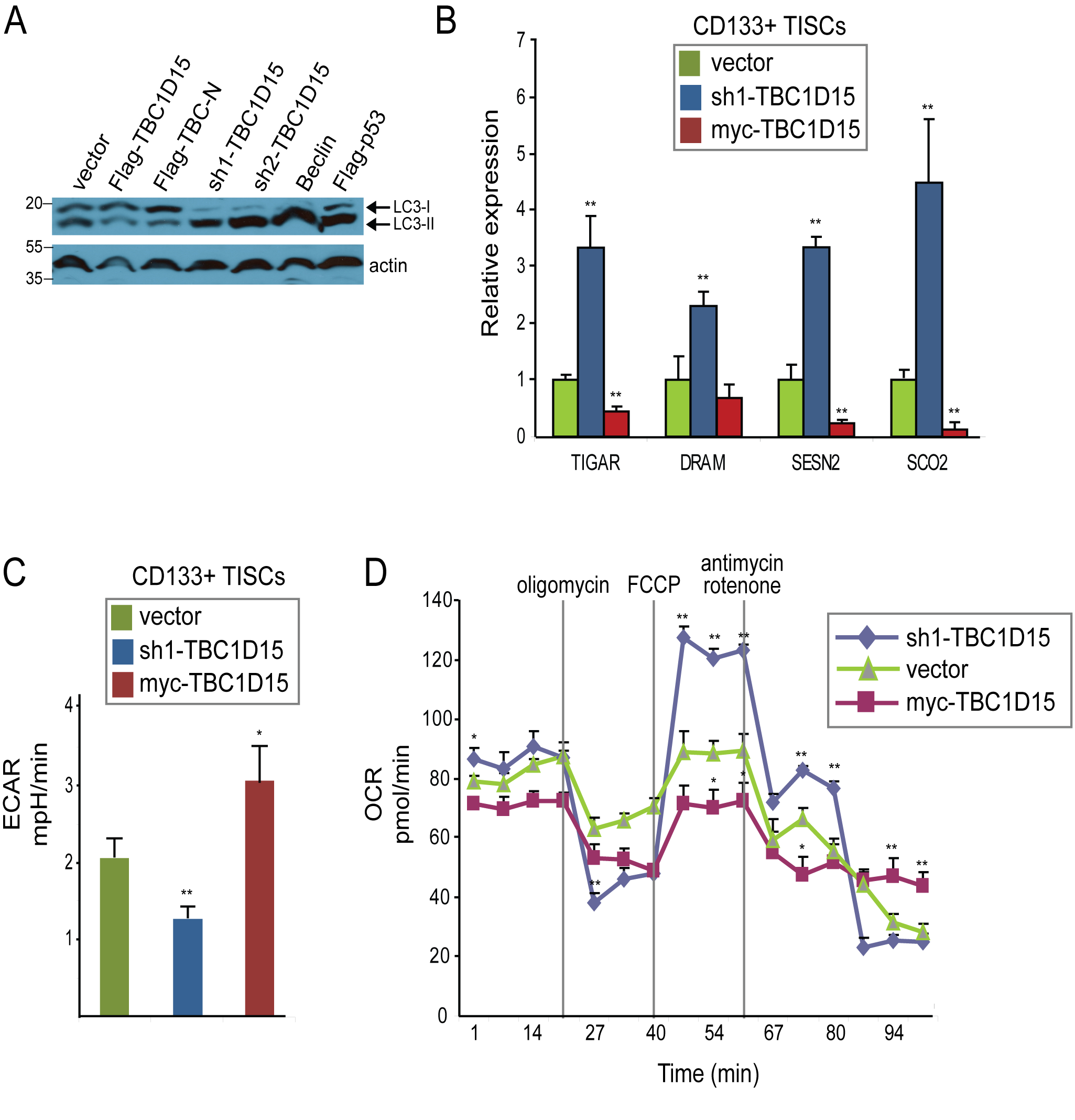

Supplement: Figure S6 — Effect of TBC1D15 on cellular metabolism. (A) Lysates prepared from murine CD133+ TISCs expressing the indicated vectors or depleted for TBC1D15 were resolved by SDS-PAGE and analyzed by immunoblotting for LC3. (B) Expression of the indicated genes was examined by qRT-PCR analysis of RNA transcripts isolated from TISCs stably expressing a control vector, sh1-TBC1D15 or myc-TBC1D15. **P<0.01 relative to vector control. (C and D) The basal extracellular acidification rate (ECAR) (C) and oxygen consumption rate (OCR) (D) were determined using the Seahorse XF-24 metabolic flux analyzer for TISCs stably expressing either control vector or myc-TBC1D15 or following depletion of TBC1D15. The OCR was measured over time in approximately 7 min intervals. The first three measurements were conducted to establish a baseline rate, followed by three measurements after the addition of oligomycin, an ATPase inhibitor. Following uncoupling of the proton gradient with FCCP, the maximum OCR rates were determined over the next three time intervals. A final series of measurements were conducted after inhibition of the mitochondrial respiratory chain with antimycin and rotenone. All experiments were conducted in triplicate. (TIF) [file pone.0057312.s006.tif]

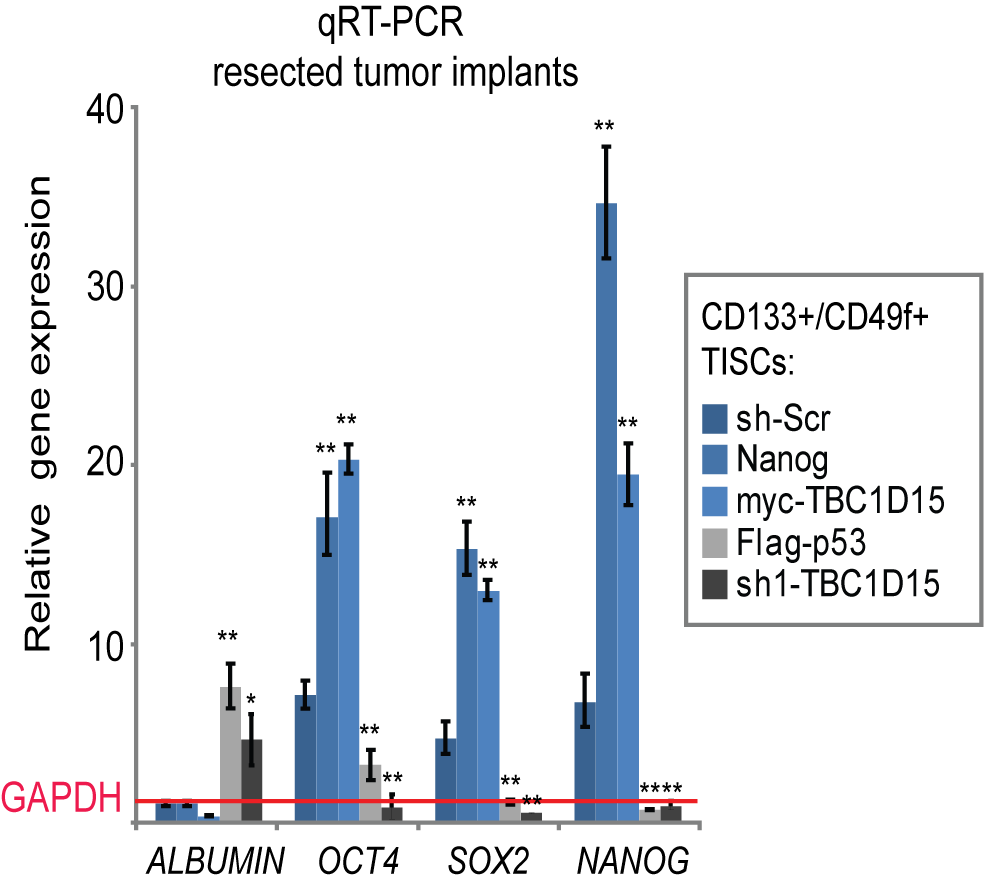

Supplement: Figure S7 — Analysis of gene expression in resected tumor implants. Implanted tumors derived from the indicated TISC lines were harvested surgically and mechanically dissociated, followed by extraction of total RNA. Expression levels of the indicated genes was determined by quantitative RT-PCR. At least three independent biological replicates were performed for each sample. Error bars represent the standard deviation. (TIF) [file pone.0057312.s007.tif]

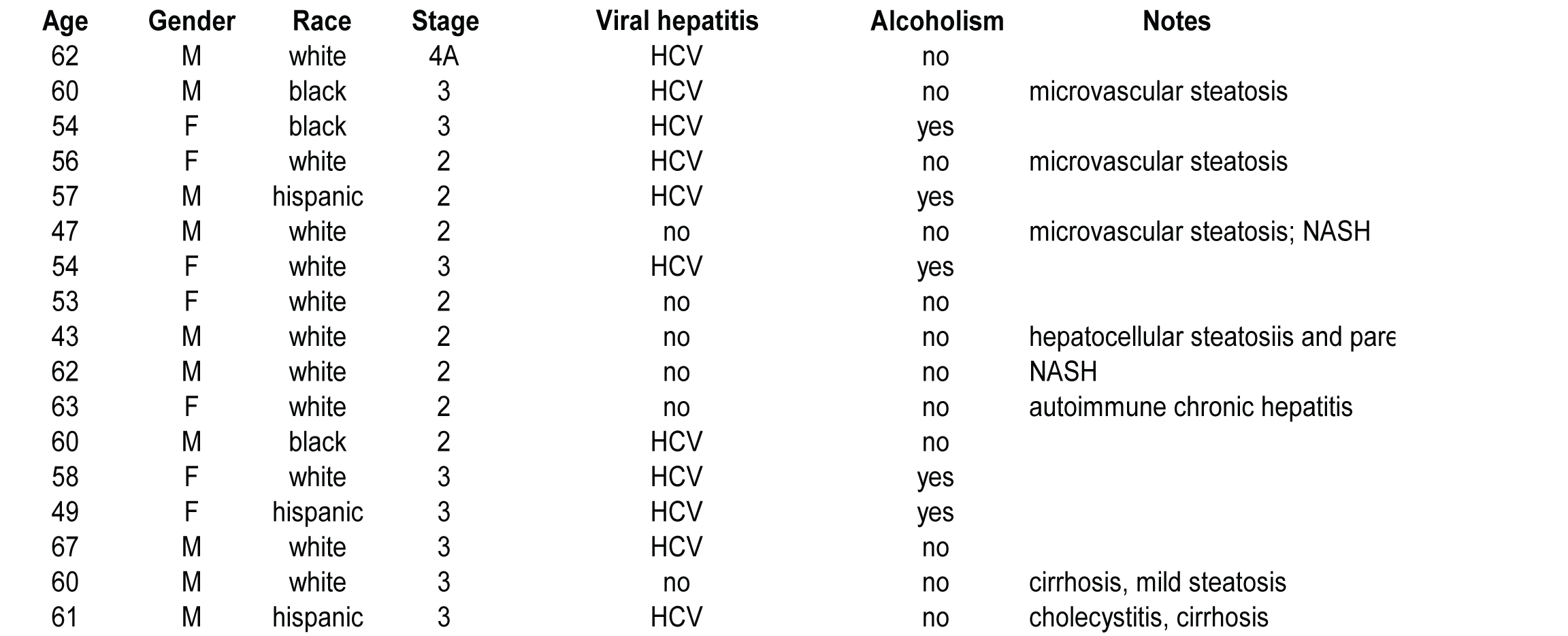

Supplement: Table S1 — Clinicopathologic features of patient tissue samples. Clinicopathologic characteristics of patient HCC tumor and matched normal tissue samples that were included as part of this study and scored for TBC1D15 immunoreactivity. NASH, non-alcoholic steatohepatitis. (TIF) [file pone.0057312.s008.tif]
